# Maize (*Zea mays* L.) productivity and profitability response to inorganic N-P nutrients, biochar and vermicompost levels in acidic soils of Northwestern Ethiopia

**DOI:** 10.1186/s12870-026-08261-y

**Published:** 2026-02-06

**Authors:** Habtamu Tadele, Tesfaye Feyisa, Lewoye Tsegaye, Sintayehu Musie

**Affiliations:** 1 Department of Natural Resource Management, College of Agriculture and Environmental Sciences, Bahir Dar University, Bahir Dar, Ethiopia; 2 Department of Natural Resource Management, Debre Markos University, Burie Campus, Burie, Ethiopia; 3 Soil and Water Research Directorate, Amhara Agricultural Research Institute, Bahir Dar, Ethiopia; 4 Crop and Horticulture Science, Department of Horticulture, Debre Markos University, Debre Markos, Ethiopia

**Keywords:** Biochar, Maize, Nitrogen, Phosphorus, Soil acidity, Vermicompost, Yield

## Abstract

**Background:**

Maize (*Zea mays* L.) is sensitive to abiotic factors that can severely diminish crop yields. The combined use of organic and inorganic sources of nutrients enhances soil fertility and crop productivity. However, site and crop-specific integration levels are essential for optimal outcomes.

**Aims:**

This study aimed to identify the optimal N/P₂O₅, biochar (BC), and vermicompost (VC) combination to improve the physiological traits, growth, yield components, and yield of maize (BH-661) under rainfed agriculture.

**Methods:**

A field experiment was conducted over two consecutive growing seasons (2023/24 and 2024/25) to identify the optimal combination of N/P₂O₅, BC, and VC for improving physiological traits, growth, yield components, and yield of maize. The experiment was arranged in a 3 × 3 × 3 RCBD design with three replicates. Treatments included three levels each of N/P₂O₅ (0/0, 120/69, 240/138 kg ha⁻¹), BC (0, 4, 8 t ha⁻¹), and VC (0, 5.02, 10.04 t ha⁻¹). Data were collected on days to 90% physiological maturity (DPM), number of leaves per plant (NLPP), plant height (PH), ear length (EL), ear number per plant (ENPP), grain number per ear (GNPE), number of rows per ear (NRPE), thousand grain weight (TGW), biological yield (BY), and grain yield (GY).

**Results:**

The analysis of variance (ANOVA) indicated that maize phenology, growth, yield components and GY were significantly different (*p* < 0.01). The highest GY (12.13 t ha⁻¹), PH (320.5 cm), and GNPE (647.11) were recorded under integrated applications, particularly the combined application of 120 kg N ha⁻¹ + 69 kg P_2_O_5_ ha⁻¹ + 8 t BC ha⁻¹ + 10.04 t VC ha⁻¹ (T24) in soils that received a uniform lime application of 0.63 t CaCO_3_ ha⁻¹. The lowest values were observed in the control (T1). Notably, 120/69 kg N/P₂O₅ ha⁻¹ + 4 t BC ha⁻¹ + 5.02 t VC ha⁻¹ (T14) consistently produced high economic yields (12.09 t ha⁻¹) and the highest net benefit (289,124 ETB ha⁻¹) with a marginal rate of return of 149.067%.

**Conclusion:**

Combining 120/69 kg N/P₂O₅ ha⁻¹, 4 t BC ha⁻¹, and 5.05 t VC ha⁻¹ (T14) is recommended to enhance maize yield and profitability in the area. Further multi-season, multi-location studies with additional data on physiological, molecular and nutritional traits are needed to validate and consolidate these findings.

## Introduction

The global challenge of sustainable food production is compounded by widespread soil fertility depletion, primarily due to insufficient nutrient management and prolonged cultivation without effective soil recovery measures [1]. Maize (*Zea mays* L.) is a crucial staple crop worldwide, particularly for millions of smallholder farmers in Sub-Saharan Africa (SSA), where it serves as a primary source of food, feed, and income. Its adaptability, high yield potential, and wide range of uses make it central to food security and rural livelihoods. However, achieving its full productivity is often hindered by declining soil fertility especially nutrient deficiencies and soil acidity which significantly limit yields and reduce farmers’ incomes [2, 3].

In tropical Africa, soil acidity and degradation are major constraint to maize production, especially in areas with moderate to high agricultural potential [4, 5]. Factors contributing to soil degradation include continuous cropping, population pressure, limited organic fertilizer use, reliance on inorganic fertilizers, erosion, and nutrient leaching [6, 7].

In Ethiopia, poor farming practices have led to significant nutrient and organic matter depletion, threatening the sustainability of agriculture [8]. Additional constraints such as drought, suboptimal agronomic practices, limited input access, and lack of acid-tolerant maize varieties further suppress yields [9, 10]. Nutrient management, particularly nitrogen (N) and phosphorus (P) fertilization, is critical to unlocking maize yield potential in Ethiopia’s diverse agro-ecologies [11]. In northwest Ethiopia’s maize zones, including Burie district, N and P are the most limiting nutrients [11]. While inorganic fertilizers (IF) are essential for modern agriculture [12], their overuse can lead to environmental problems such as soil acidification, greenhouse gas emissions, and pollution [13, 14]. Continuous nitrogen fertilizer application accelerates soil acidification through nitrification, lowering pH, and restricting nutrient availability.

To reduce these negative impacts, integrating organic amendments like BC and VC with IF is increasingly recommended. Organic fertilizers (OF) improve soil fertility and health by enhancing physical, chemical, and biological properties [15]. Among these, biochar, is a stable carbon-rich product from biomass pyrolysis, has shown promise in improving soil organic matter, nutrient retention, and crop productivity, particularly in degraded tropical soils [16–18]. Biochar is effective for acidity remediation, sometimes outperforming lime due to additional benefits such as nitrogen retention and carbon sequestration [19, 20]. Moreover, vermicompost, produced by earthworm-mediated decomposition, enriches soil nutrients, improves structure, and stimulates microbial activity, aiding soil restoration on degraded lands [21, 22]. Combining BC and VC stabilizes organic matter and reduces nutrient losses, further enhancing soil fertility management [23].

In Ethiopia’s maize belt zones, soil acidity is a significant yield-limiting factor. Maize prefers soils with pH around 5.5–5.7; highly acidic soils (pH ≤ 5.0) inhibit growth due to toxic aluminum and manganese levels and nutrient deficiencies [24, 25]. Integrated soil fertility management (ISFM), which combines organic and inorganic nutrient sources, is critical to optimize yield and sustain soil health [26, 27]. Despite ISFM’s potential to combat nutrient depletion and boost productivity across SSA, adoption remains limited by socio-economic and logistical challenges [28].

Hence, high fertilizer costs and limited availability underscore the need for efficient use of organic inputs like VC and BC alongside reduced IF rates [29]. Partial replacement of IF with organic amendments improves soil quality and reduces environmental harm [30]. Declining nutrient use efficiency (NUE), especially for nitrogen, caused by imbalanced fertilizer use, remains a challenge [31]. Studies show that combining lower rates of IF with organic amendments can increase NUE while maintaining or improving yields [32, 33]. This synergy also improves soil physical properties like bulk density and nutrient retention, enhancing nutrient uptake [34, 35]. Despite these benefits, sustainable practices and ISFM remain under used in Ethiopia. Agricultural sustainability means maintaining or increasing productivity over time through sound land and nutrient management [36].

In the Burie district, maize production relies heavily on inorganic fertilizers without organic amendments such as BC or VC made from local residues like maize cobs, limiting soil nutrient restoration and productivity gains. Recommended nitrogen and P₂O₅ rates (240 kg N and 138 kg P₂O₅ ha⁻¹) and plant spacing exist [37], but integrating organic amendments could improve physiological performance and economic yield.

While the benefits of IF and OF are known, there is limited understanding of how N, P₂O₅, BC, and VC interact in very acidic Ethiopian highland soils, including Burie district. Previous studies have not thoroughly evaluated their combined effects over multiple seasons. This study aims to identify the optimal N/P₂O₅, BC, and VC combination to enhance maize growth, physiological performance, yield traits, and yield under rainfed agriculture. Specifically, it aims to (i) determine optimal as well as economical organic and inorganic nutrient combinations, and (ii) examine their effects on maize physiological, growth, and yield-related traits, and yield of maize. Therefore, unified use of inorganic and locally available organic sources of nutrients was optimized in acidic soils under rainfed conditions of two years study in Burie district, Ethiopia.

## Materials and methods

### Experimental location and treatments

**Fig. 1:**
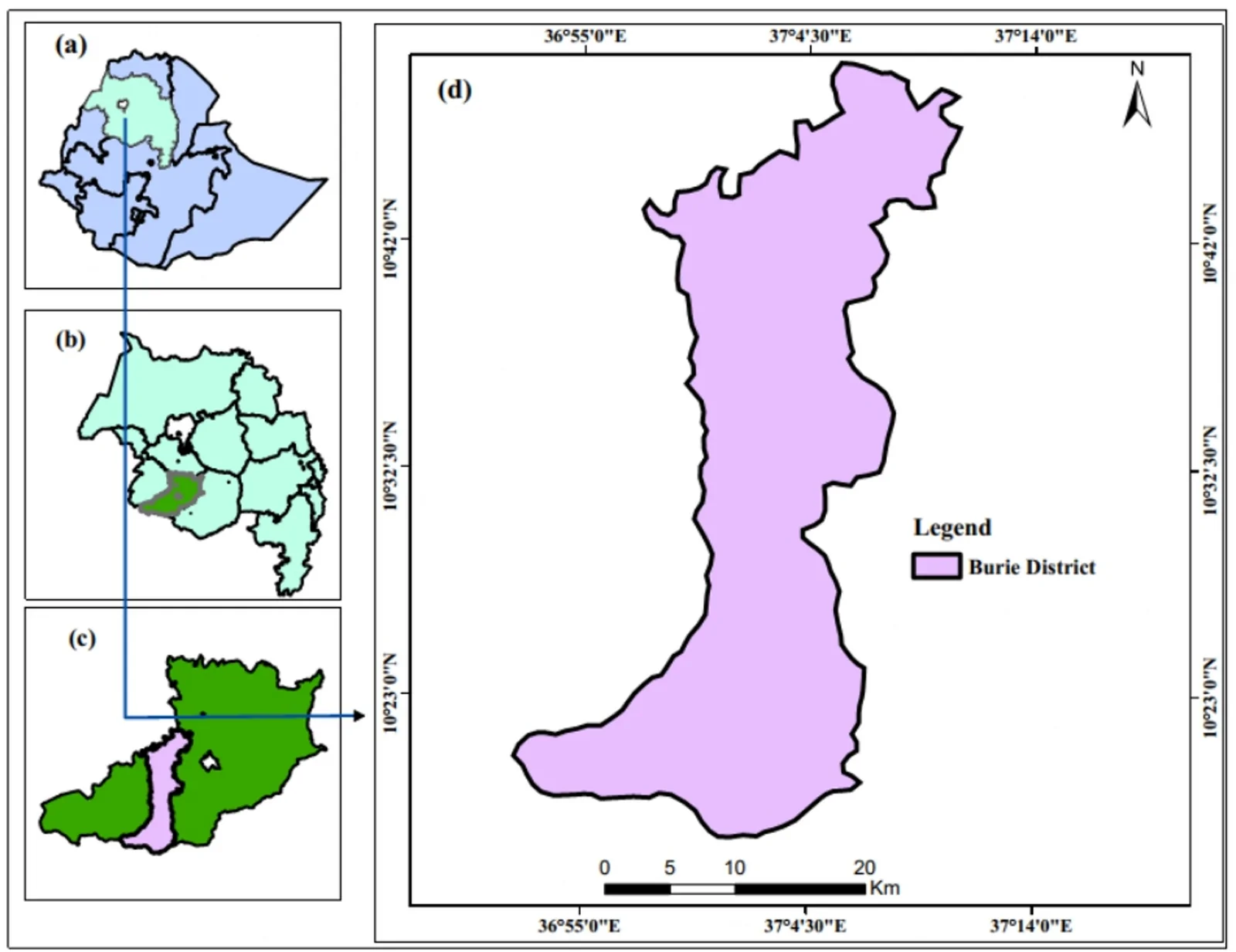
Study area map showing: (**a**) Amhara Region within Ethiopia, (**b**) West Gojjam Zone within the Amhara Region, (**c**) Burie District within West Gojjam Zone, and (**d**) the specific study site in Burie District (Habtamu Tadele, ArcGIS, 2023)

**Fig. 2:**
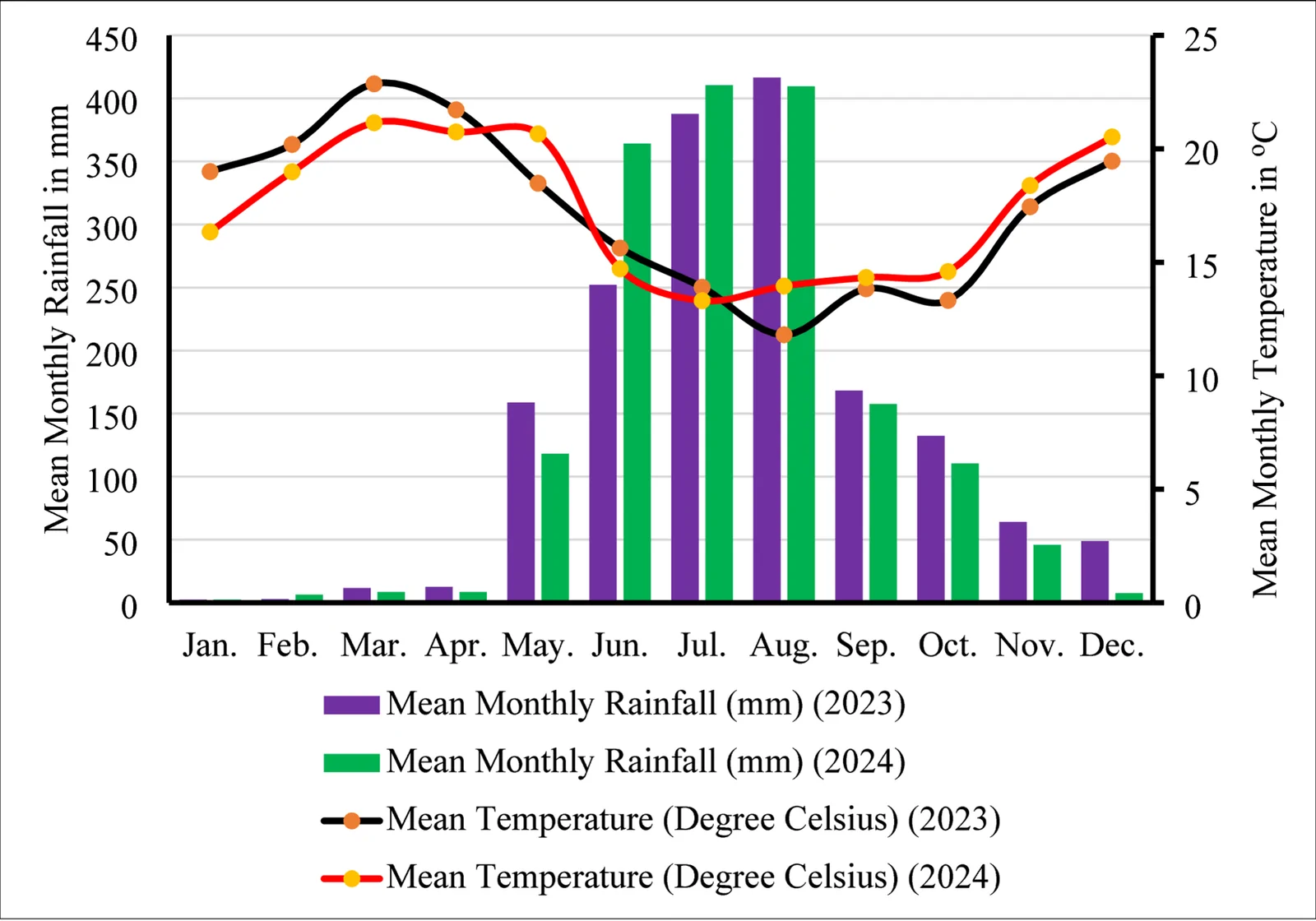
Mean monthly rainfall (mm) and mean monthly temperatures (°C) of the study area recorded from 2023/24-2024/25

Maize variety (BH-661), which is well adapted in the area was used in this study over two consecutive growing seasons (2023-24 & 2024-25). The seed was purchased from Certified Maize Seed Multiplication center, Burie district branch. During the 2023-24 & 2024-25 cropping seasons, the market prices were 1,100 and 1,400 Ethiopian Birr (ETB) (approx. USD 20.20 and USD 24.45, respectively) per 12.5 kg package of chemically coated seed. The seed rate was 42.61 kg ha⁻¹. The average market price of a 12.5 kg seed package over two years was 22.33 USD. Therefore, the total cost of 42.61 kg of seed was USD 76.10. The field experiments were conducted at Debre Markos University, Burie Campus research site (10° 23’-10° 42’ N, 36° 55’-37° 14’ E, 2146–2148 m above sea level) (Fig. 1). The area experiences a sub-humid climate with an average annual rainfall of 1654 mm and mean temperatures ranging from 14.4 °C to 20.16 °C (Fig. 2). The mixed crop-livestock system is the main land use system in the study area. Prior to the establishment of the field experiment, the site had been cropped with maize and faba bean (*Vicia faba* L.), in alternating years. These previous crops may have influenced baseline soil properties, particularly in terms of nitrogen availability and organic matter. Initial soil sampling was conducted to account for these background effects.

### Soil sampling and laboratory procedures

Soil sampling was conducted before sowing using both a soil auger and a core sampler to assess the soils physical and chemical characteristics. On February 10, 2023, prior to planting, ten subsamples were collected from the 0–20 cm depth using an auger sampler and thoroughly mixed to obtain one composite sample for chemical analysis. Similarly, ten undisturbed samples were collected from the 6 cm depth using a core sampler with a 5 cm internal diameter (i.d.) for determining bulk density (BD), total soil porosity (TSP), and gravimetric moisture content (GMC). All composite and individual soil samples were properly labeled. The labels were placed both inside and outside the plastic bags and were transported to the soil laboratory of Bahir Dar University Soil Physics and Plant Analysis.

Standard laboratory procedures were followed: soil pH was determined using a 1:2.5 soil-to-water ratio [38]; Total Nitrogen (TN) was analyzed by the Kjeldahl method [39]; Organic carbon (OC) by the Walkley and Black method [40]; Organic matter (OM) was calculated by multiplying OC by 1.724 [40]; available Phosphorus (av.P) was extracted using the Mehlich-III method [41]; exchangeable Al and H: (1 N KCl extraction); Exc.Ac was determined using a 1 N KCl extraction followed by titration [42].

### Soil physico-chemical properties of the experimental site

Pre-experimental soil properties are described in Table 3. The BD and SP of the experimental soil were 1.41 g cm⁻³ and 46.49%, respectively. The GMC at the time of sampling, reflecting natural field conditions, was 14%. This GMC level is below the optimal range for active plant growth but slightly above the wilting point for study sandy clay loam soils. The pH was 4.94, classifying it as very strongly acidic. Chemical analysis showed low fertility, with OC at 2.11%, TN at 0.25% and av.P at 11.69 mg kg⁻¹, indicating poor soil fertility conditions (Table 3). The soil at the study site was classified as sandy clay loam texture, with 50% sand, 36% clay, and 14% silt. Bulk density was slightly above the ideal threshold (1.41 g cm⁻³), and porosity was suboptimal at 46.79%.

## Production and characterization of biochar and vermicompost

### Biochar production, characterization and application

**Fig. 3:**
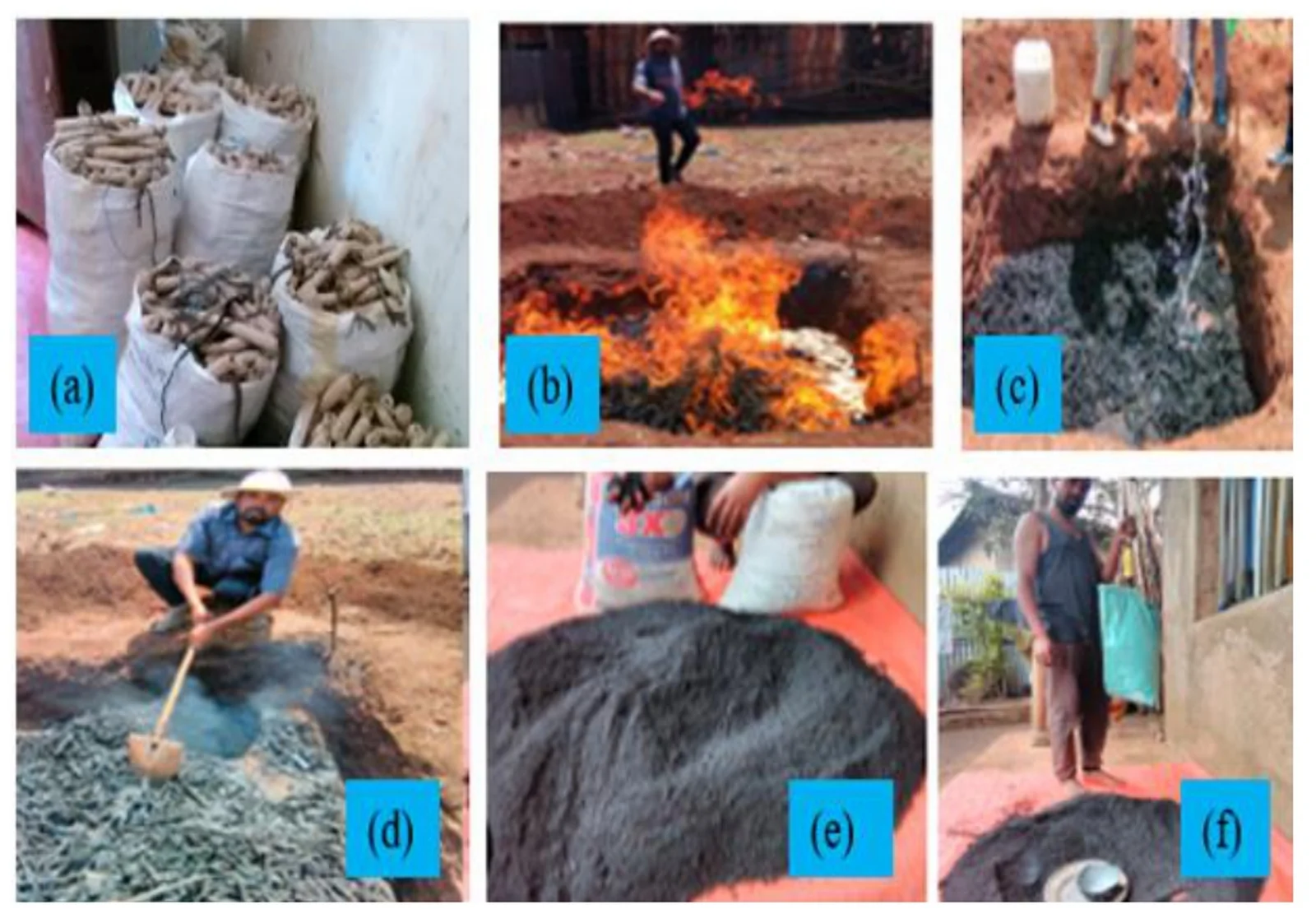
Illustration of maize cob BC preparation in the soil pyrolyzer using open pit method. Maize cob (**a**), 2 m length × 1.5 m width × 1 m depth soil pyrolyzer pit and burning the maize cob in pit at midday or at sunny time (**b**), quenching the pyrolyzed corn cob with water after burning (**c**), cooling BC (**d**), BC ready for crashing (**e**), final BC to be applied weighed its mass for treatment (**f**)

Biochar was produced using open-pit pyrolysis of maize cobs, with materials quenched before full combustion as illustrated in Fig. 3 below. Physicochemical properties of BC and VC are described in Table 3. The carbonization of the maize cob is anticipated to take place beneath the flames under low oxygen since the flames consume all of the feedstock, forming a pyrolysis chamber, much like the open kiln method of biochar preparation [43]. The yield of BC was calculated based on the mass of feedstock and final product as described by [44]. The weight of BC in kg produced from the 212.34 kg and 208.49 kg air-dried feedstock was determined as the BC yield using [44]. Biochar samples were sieved and analyzed for pH, TN, OC, and av.P following the method of [45].

Activities and cost of production for BC are presented in Table 1. For the 8.25 m² experimental plot, 6.6 kg of BC was applied, representing the full recommended rate of 8 t BC ha⁻¹, while 3.3 kg corresponded to the 4 BC t ha⁻¹ application rate. In 2023, 212.34 kg of dry maize cob biomass was utilized to produce 89.1 kg of BC for the experimental soils, whereas in 2024, 208.49 kg of dry maize cob biomass yielded the same quantity of BC. The observed variation is likely attributable to differences in maize cob characteristics and moisture content between the two years. The BC yield percentages were 41.96% and 42.73% for 2023 and 2024, respectively, resulting in an average dry biomass weight of 210.42 kg and a mean BC yield of 42.35%.

On a hectare basis in 2023, 19,065.32 kg of maize cob dry weight (190.65 quintals) was required to produce 8,000 kg of BC, representing the full recommended BC application rate for maize. The BC yield percentage in 2023 was 41.96%. Similarly, in 2024, 18,719.64 kg of maize cob dry weight (187.2 quintals) was needed to obtain 8,000 kg of BC, with a BC yield percentage of 42.74%. The average maize cob dry weight over the two years was 18,892.48 kg (188.92 quintals), with a mean BC yield of 42.34%. On average, 1 quintal of dry maize cob biomass weighed 38.86 kg and yielded 16.46 kg of BC. To produce 8 t of BC, 486.02 quintals of dry maize cob biomass are required. The market price of 1 quintal of maize cob was 250 ETB in 2023 and 150 ETB in 2024, with a mean price of 200 ETB. In 2023-24 and 2024-25 cropping season, USD 1 was 53.87 and 56.32 ETB, respectively. The unit price for 1 t of BC was calculated by dividing total cost of production 41,280 ETB ha⁻¹ by mean of BC used across two years (8 t ha⁻¹), resulting in 5,160 ETB.

The BC produced fulfilled the criterion for carbon content (< 45%). The produced BC had a carbon content of 64.62%, the pH of 9.88, TN (1.11%), and av.P (46.5 mg kg⁻¹). A biochar OC content of 64.62% from maize cobs is considered high. This value aligns with findings from various studies on BC produced from maize cobs. The slight increase in av. P content of BC from 44.71 mg kg⁻¹ in 2023 to 48.36 mg kg⁻¹ in 2024 can be attributed to variations in pyrolysis conditions and feedstock characteristics. Differences in temperature control during pyrolysis or in the nutrient composition of maize cobs-resulting from climatic, varietal, or soil nutrient differences-may have contributed to this change. Additionally, annual variation in agronomic practices and BC processing could influence phosphorus retention and availability. These factors, individually or in combination, likely explain the observed year-to-year difference. Biochar yield ranged between 41.96% and 42.73% from the feedstock across both years. The physicochemical properties of the produced BC and VC (See Table 3) indicate they are nutrient-rich amendments suitable for soil application.

For instance: a study by [46] reported a fixed carbon content of 60.5% in maize cob biochar produced at 300 °C, while [47] reported 60.69%. The TN content of the biochar in the present study (1.11%) was higher than the 0.82% reported by [46] for maize cob biochar produced at the same temperature. In summary, the findings demonstrate that BC possesses high quality and serves as a promising source of nutrients and liming materials.

### Vermicompost production, characterization and application

**Fig. 4:**
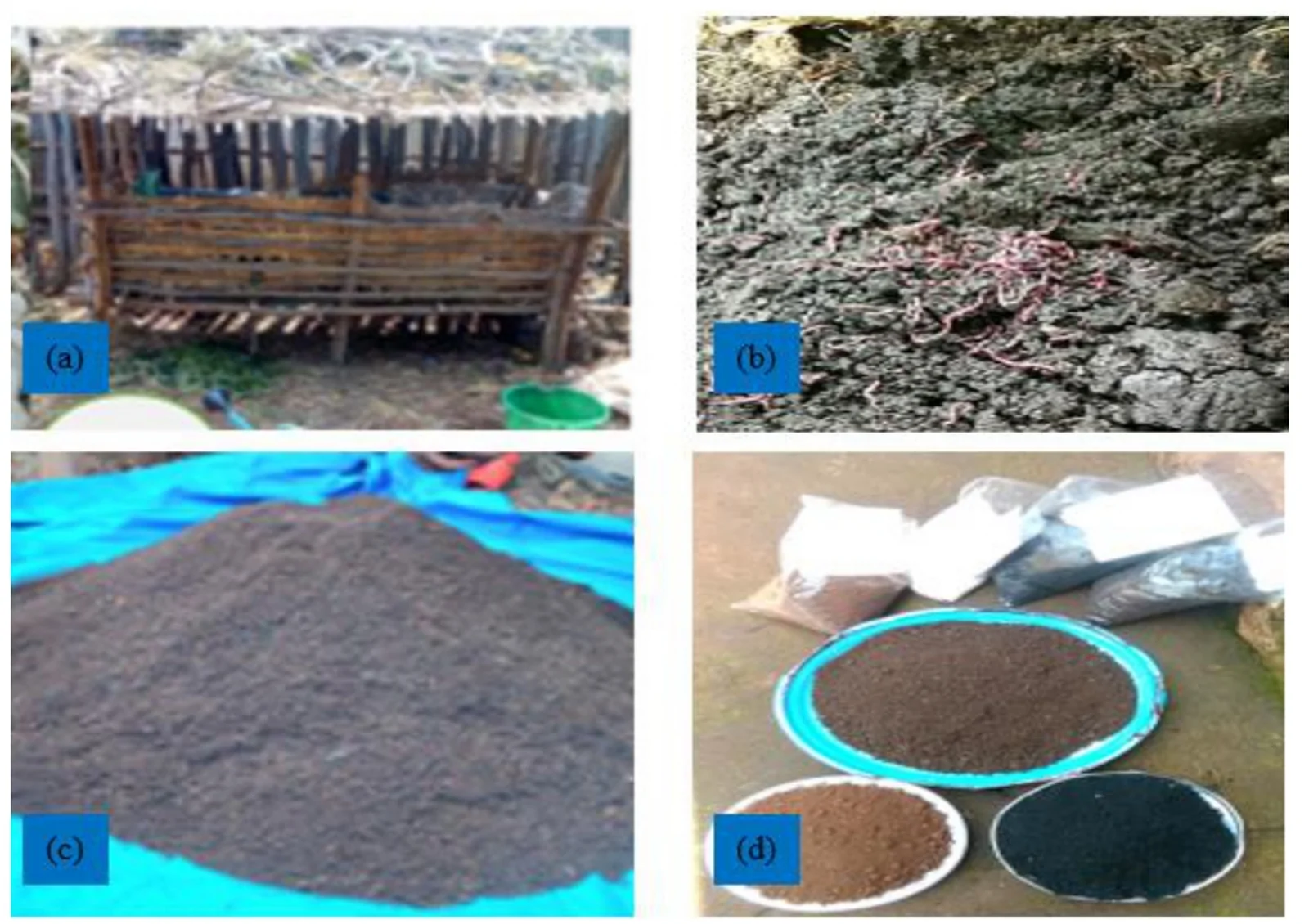
Illustrations of VC preparation by making VC earthworms’ bin for treatments. Earthworms bin (2 m length, 0.6 m width and 0.5 m height) and watering for about 15 days before adding worms with old cow dung, green leaves used after chopping (*Vernonia auriculifera* Hiern*)* (**a**), Incorporated *Epigeic fetida* worm’s (**b**), harvested VC (**c**) and sampled VC, BC and experimental soils analyzed (**d**)

Vermicompost was prepared in earthworm bins using chopped shrub species local name of Gengerita (*Vernonia auriculifera* Hiern), cow dung, and topsoil, pre-composted for 14 days, and inoculated with earthworm (*Eisenia fetida)* as illustrated Fig. 4. The VC was harvested after 60 days, dried, sieved, and analyzed for TN, OC, and av.P following standard procedures. The predetermined amount of VC was applied at sowing in rows in plots receiving this treatment according to the previous randomization set [11]. A dry weight of old cow dung weighed 25.67 kg was priced at 20.59 ETB. The unit price for 1 t of VC was calculated by dividing total cost of production 21, 388.19 ETB ha⁻¹ by mean VC used across two years (10.04 t), resulting in 2,130.29 ETB VC t⁻¹. Biomass conversion ratios indicate that 1 quintal of old cow dung yields approximately 20.8 kg of VC.

VC exhibited a slightly alkaline pH of 7.8, high OC (10.9%), and very high TN content (2.42%). It also had a narrow C: N ratio of 4.5, and av.P of 48.3 mg kg⁻¹, indicating high nutrient quality (Table 3). The substantial increase in SOC from 8.29% in 2023 to 13.56% in 2024 under VC application can be attributed to the cumulative and residual effects of organic matter inputs. VC contributes a high amount of stable OM, which may not fully decompose in a single season, leading to year-on-year buildup. Additionally, favorable climatic conditions and enhanced plant biomass in 2024 may have further contributed to SOC through increased root turnover and residue return.

**Table 1:** Maize cob BC and VC activity by cost of production values in ETB (USD 1 = 55.09 ETB) in 2023-24 & 2024-25 per ha basis

Biochar				
Activity	Unit	Quantity	Unit Cost	Total cost (ETB)
Maize cob collection	Quintal	188.92	200	37,784
Transportation	Quintal	188.92	5.00	944.60
Pyrolysis labor	Persons-day	2 × 2	225	900.00
Quenching with water	Liter	200	1.75	351.00
Sieving and Packaging	Persons-day	2 × 2	225	900.00
Tools	--	---	--	400.00
Total	---	----	---	41,280 ETB (USD 749.32)
Vermicompost				
Collection of organic materials	Quintal	482.65	20.59	9,937.76
Preparation of raw materials	Quintal	482.65	10.00	4820.00
Construction of earthworm bins	Persons-day	1 × 2	225.00	450.00
Purchase or maintenance of earthworms	Kilogram	3	234.14	702.43
Moisture and temperature control (watering, shading)	Persons-day	1 × 10	225.00	2250.00
Mixing during composting	--	---	--	400.00
Sieving and packaging	Persons-day	2 × 2	225	900.00
Transportation	Quintal	482.65	4.00	1928.00
Total	---	----	---	21,388.19 ETB (USD 388)

The local quintal used here differs from the standard metric quintal of 100 kg

### Calculation of VC nitrogen equivalence (NEvc)

In both cropping seasons, the TN content of VC was measured prior to application. The nitrogen equivalence of VC (NEvc) was calculated using the formula: NEvc = 10 × TN, where NEvc is the nitrogen equivalence VC t⁻¹, TN is the total nitrogen (%) per 100 kg of VC, and 10 is the conversion factor used to scale the nitrogen content from 100 kg to 1 tonne.

### Experimental design, treatments, field layout, and procedure

**Fig. 5:**
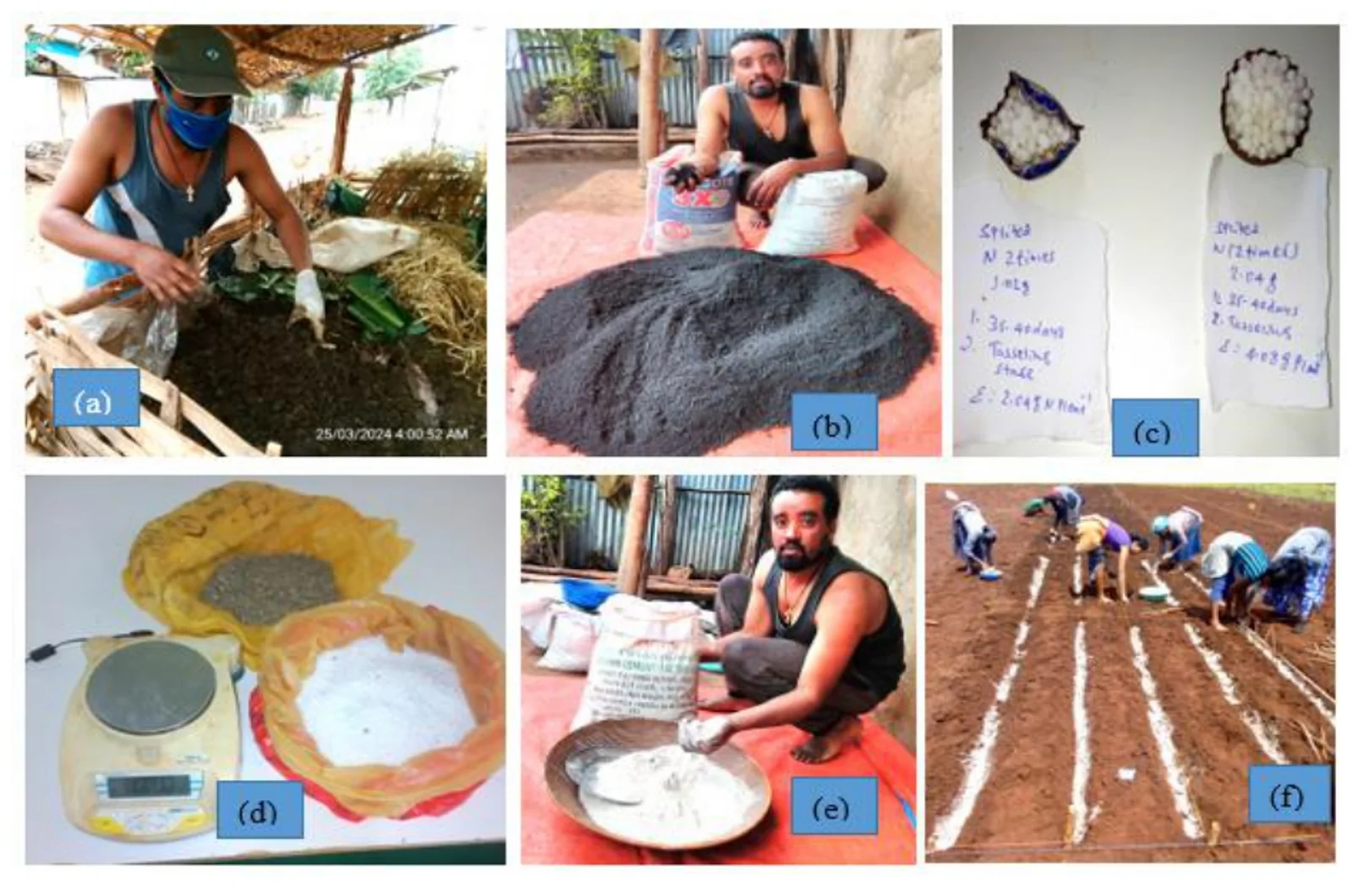
Illustrations of treatment inputs and sowing: (**a**) vermicompost, (**b**) maize cob biochar, (**c**) calibrated urea, (**d**) inorganic urea and NPSB fertilizers, (**e**) lime (CaCO₃), and (**f**) uniform lime application in rows at sowing. (Photo by Habtamu Tadele, 2024)

**Fig. 6:**
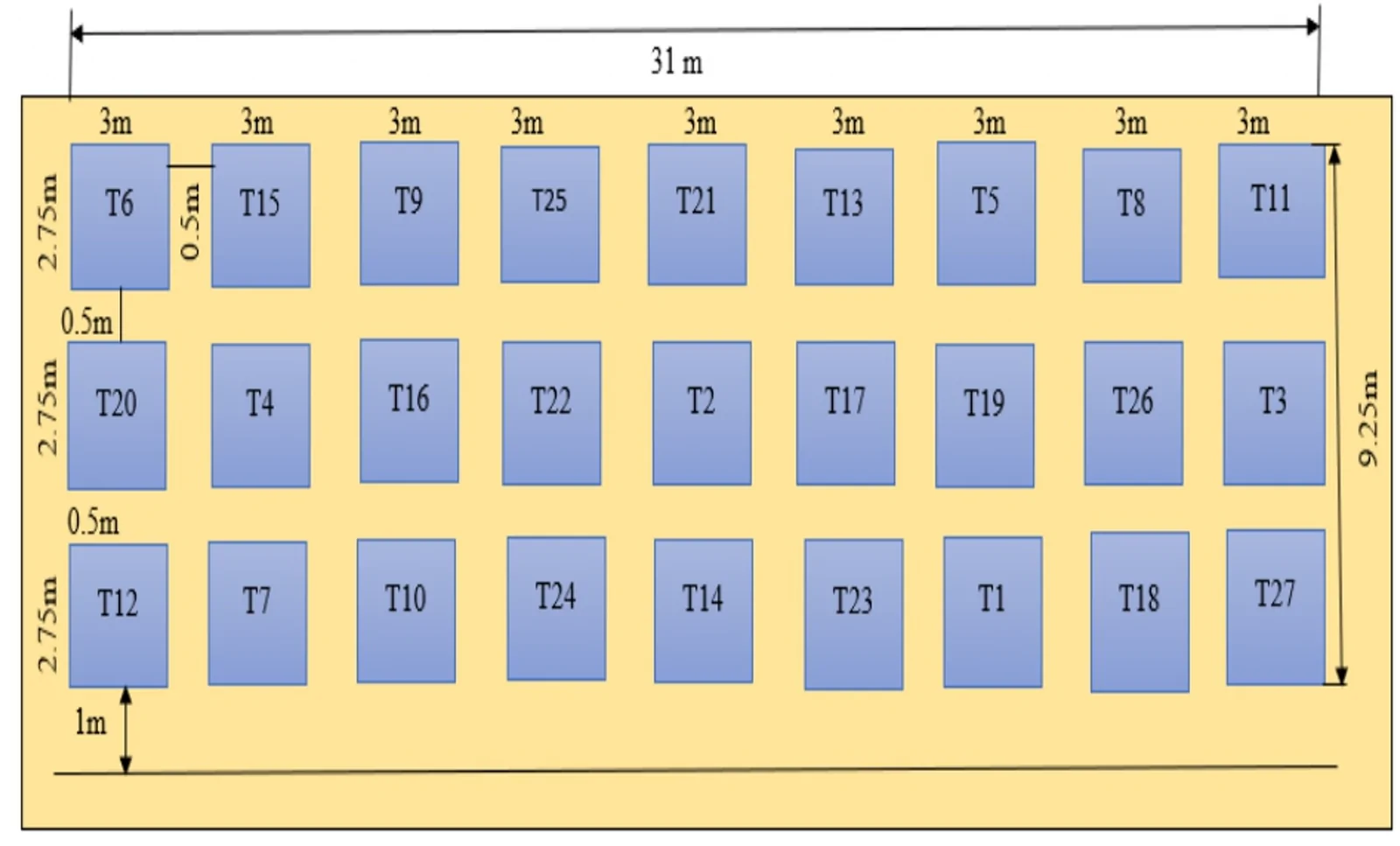
Maize field layout map showing one replication of the 3 × 9 factorial randomized complete block design (27 treatments folded into 3) used for experiments at Debre Markos University Burie Campus research site in 2023-24 and 2024-25

This experiment was laid out as a RCBD with three replications (Table 2; Figs. 5 and 6). The BH-661 variety was used as a test crop at a seeding rate of 42.61 kg ha⁻¹. The study involved three factors: three levels of N/P_2_O_5_ (0/0, 120/69, 240/138 kg ha⁻¹), three levels of BC (0, 4 and 8 t ha⁻¹) and three levels of VC (0, 5.02 and 10.04 t ha⁻¹) to have a total of 27 treatments that were arranged in 3 × 3 × 3 factorial combinations (Fig. 6).

On the basis of exchangeable acidity (Exc. Ac) of experimental soils, uniform lime rate equivalent to 25% of the recommended dose was applied to all plots. This recommendation is based on the unpublished 2023 extension manual from the Burie district Agricultural Office, developed for cereal crops like maize following evaluation of soil pH and exchangeable acidity (Exc. Ac) levels. Lime requirement was calculated following the method described by Kamprath [48], using exchangeable acidity as a base factor.

$$\:LR\:\left({cmol}_{c}\:{kg}^{-1}\:soil\right)=lf\times\:\:ExcAc\:\left({cmol}_{c}\:{kg}^{-1}\:soil\right)\:\:\:\:\:\:\:\:\:\:\:\:\:\:\:\:\:\:\:\:\:\:\:\:\:\:\:\:\:\:\:\:\:\:\:\:\:\:\:\:\:\:\:\:\:\:\:\:\:\:\:\:\:\:\:\:\:\:\:\:\:\:\left(1\right)$$

Abbreviations: LR, lime requirements (CaCO_3_), Exc. Ac, the initial exchangeable acidity of the soil (H^+^ and Al^3+^), lf, the lime factor, which is 1.5 times the initial Exc. Ac (cmol_c_ kg⁻¹) will be enough to reduce the acidity saturation to at most 15%, which is to be considered to be a threshold below which most crops are not affected by acidity. Accordingly, lime was applied in rows at sowing uniformly [11, 49].

Nitrogen was applied in split doses from urea and NPSB (nitrogen, phosphorus, sulfur and boron), while phosphorus was applied fully at planting from NPSB blended IF. The Full recommended inorganic nitrogen (240 kg ha⁻¹) and P_2_O_5_ (138 kg ha⁻¹) for a maize plant population of 90, 909 (55 cm between rows × 20 cm within plants) was used as described by [37]. Each plot had a gross area of 8.25 m² and a net harvest area of 3.63 m². All agronomic practices were applied uniformly across all treatments following the recommendation practices described by [11, 50].

**Table 2:** Combination of treatments used in the field experiment (2023-24 & 2024-25)

Treatments	Description
T1	Control (0.63 t CaCO_3_ ha⁻¹)
T2	5.02 t VC ha⁻¹ + 0.63 t CaCO_3_ ha⁻¹
T3	10.04 t VC ha⁻¹ + 0.63 t CaCO_3_ ha⁻¹
T4	120/69 kg N/P_2_O_5_ ha⁻¹ + 0.63 t CaCO_3_ ha⁻¹
T5	120/69 kg N/P_2_O_5_ ha⁻¹ + 5.02 t VC ha⁻¹ + 0.63 t CaCO_3_ ha⁻¹
T6	120/69 kg N/P_2_O_5_ ha⁻¹ ^1^ + 10.04 t VC ha⁻¹ + 0.63 t CaCO_3_ ha⁻¹
T7	240 kg N ha⁻¹ + 138 kg P_2_O_5_ ha⁻¹ + 0.63 t CaCO_3_ ha⁻¹
T8	240 kg N ha⁻¹ + 138 kg P_2_O_5_ ha⁻¹ + 5.02 t VC ha⁻¹ + 0.63 t CaCO_3_ ha⁻¹
T9	240 kg N ha^− 1^ + 138 kg P_2_O_5_ ha⁻¹ + 10.04 t VC ha⁻¹ + 0.63 t CaCO_3_ ha⁻¹
T10	4 t BC ha⁻¹ + 0.63 t CaCO_3_ ha⁻¹
T11	4 t BC ha⁻¹ + 5.02 t VC ha⁻¹ + 0.63 t CaCO_3_ ha⁻¹
T12	4 t BC ha⁻¹ + 10.04 t VC ha⁻¹ + 0.63 t CaCO_3_ ha⁻¹
T13	120 kg N ha⁻¹ + 69 kg P_2_O_5_ ha⁻¹ + 4 t BC ha⁻¹ + 0.63 t CaCO_3_ ha⁻¹
T14	120 kg N ha⁻¹ + 69 kg P_2_O_5_ ha⁻¹ + 4 t BC ha⁻¹ + 5.02 t VC ha⁻¹ + 0.63 t CaCO_3_ ha⁻¹
T15	120 kg N ha⁻¹ + 69 kg P_2_O_5_ ha⁻¹ + 4 t BC ha⁻¹ + 10.04 t VC ha⁻¹ + 0.63 t CaCO_3_ ha⁻¹
T16	240 kg N ha⁻¹ + 138 kg P_2_O_5_ ha⁻¹ + 4 t BC ha⁻¹ + 0.63 t CaCO_3_ ha⁻¹
T17	240 kg N ha⁻¹ + 138 kg P_2_O_5_ ha⁻¹ + 4 t BC ha⁻¹ + 5.02 t VC ha⁻¹ + 0.63 t CaCO_3_ ha⁻¹
T18	240 kg N ha⁻¹ + 138 kg P_2_O_5_ ha⁻¹ + 4 t BC ha⁻¹ + 10.04 t VC ha⁻¹ + 0.63 t CaCO_3_ ha⁻¹
T19	8 t BC ha⁻¹ + 0.63 t CaCO_3_ ha⁻¹
T20	8 t BC ha⁻¹ + 5.02 t VC ha⁻¹ + 0.63 t CaCO_3_ ha⁻¹
T21	8 t BC ha⁻¹ + 10.04 t VC ha⁻¹ + 0.63 t CaCO_3_ ha⁻¹
T22	120 kg N ha⁻¹ + 69 kg P_2_O_5_ ha⁻¹ + 8 t BC ha⁻¹ + 0.63 t CaCO_3_ ha⁻¹
T23	120 kg N ha⁻¹ + 69 kg P_2_O_5_ ha⁻¹ + 8 t BC ha⁻¹ + 5.02 t VC ha⁻¹ + 0.63 t CaCO_3_ ha⁻¹
T24	120 kg N ha⁻¹ + 69 kg P_2_O_5_ ha⁻¹ + 8 t BC ha⁻¹ + 10.04 t VC ha⁻¹ + 0.63 t CaCO_3_ ha⁻¹
T25	240 kg N ha⁻¹ + 138 kg P_2_O_5_ ha⁻¹ + 8 t BC ha⁻¹ + 0.63 t CaCO_3_ ha⁻¹
T26	240 kg N ha⁻¹ + 138 kg P_2_O_5_ ha⁻¹ + 8 t BC ha⁻¹ + 5.02 t VC ha⁻¹ + 0.63 t CaCO_3_ ha⁻¹
T27	240 kg N ha⁻¹ + 138 kg P_2_O_5_ ha⁻¹ + 8 t BC ha⁻¹ + 10.04 t VC ha⁻¹ + 0.63 t CaCO_3_ ha⁻¹

*Abbreviations*: *T* treatments, *BC* biochar, *VC* vermicompost

**Table 3:** Pre-experimental soil, maize cob BC and VC physico-chemical characteristics

Soil parameters		Value		Status	Critical level			Reference
Soil physical Properties								
Sand (%)		50		---	---			
Silt (%)		14		---	---			
Clay (%)		36		---	---			
Textural class		SC		---	---			
Bulk Density (g cm^− 3^)		1.41			< 1.40			[51]
Soil porosity (%)		46.79		Low	> 50%			[52]
Moisture content (%)		14		---				
Chemical Properties								
Exc. H (cmol_c_ kg⁻¹)		0.88		---	---			
Exc. Al (cmol_c_ kg⁻¹)		0.8		---	---			
Exc. Ac (cmol_c_ kg⁻¹)		1.68		Low	2 cmol_c_ kg^− 1^			[11]
Soil pH (1:2.5, water)		4.94		VSA	6.6–7.3			[53]
C-organic (%)		2.11		Low	4.0–10			[54]
N-total (%)		0.25		Low	0.5			[55]
C: N		8.44		----	---			
SOM (%)		3.63		Optimum	3.0–7.0			[53]
av.P (mg kg⁻¹)		11.69		Very low	30–80			[53]
Nutrient content of vermicompost and maize cob biochar								
Parameters	Vermicompost				Maize cob biochar			
	2023	2024	Mean	Rate	2023	2024	Mean	
pH (1:20, water)	7.78	7.82	7.8	SAL	10.14	9.63	9.88	[56]
OC (%)	8.29	13.56	10.9	High	65.46	63.78	64.62	[46, 47]
TN (%)	2.16	2.68	2.42	VH	0.36	1.86	1.11	[54]
av.P (mg kg⁻¹)	46.71	49.89	48.3	High	44.71	48.36	46.5	[54]
Colour	Dark grey to black							

*Abbreviations*: *pH*, *soil*, *BC* and *VC* reaction, *SAL *slightly alkaline, *VH *very high, *SCL *sandy clay, *SAL *Strongly alkaline, *VSA *very strongly acidic, *Exc.Ac *exchangeable acidity, *Exc.Al *exchangeable aluminum, *Exc.H *exchangeable hydrogen, *SOC *soil organic carbon, *TN *total nitrogen, *av.P* available phosphorus

## Data collections

### Crop parameter data collection and measurement

#### Crop phenology and growth traits

**Fig. 7:**
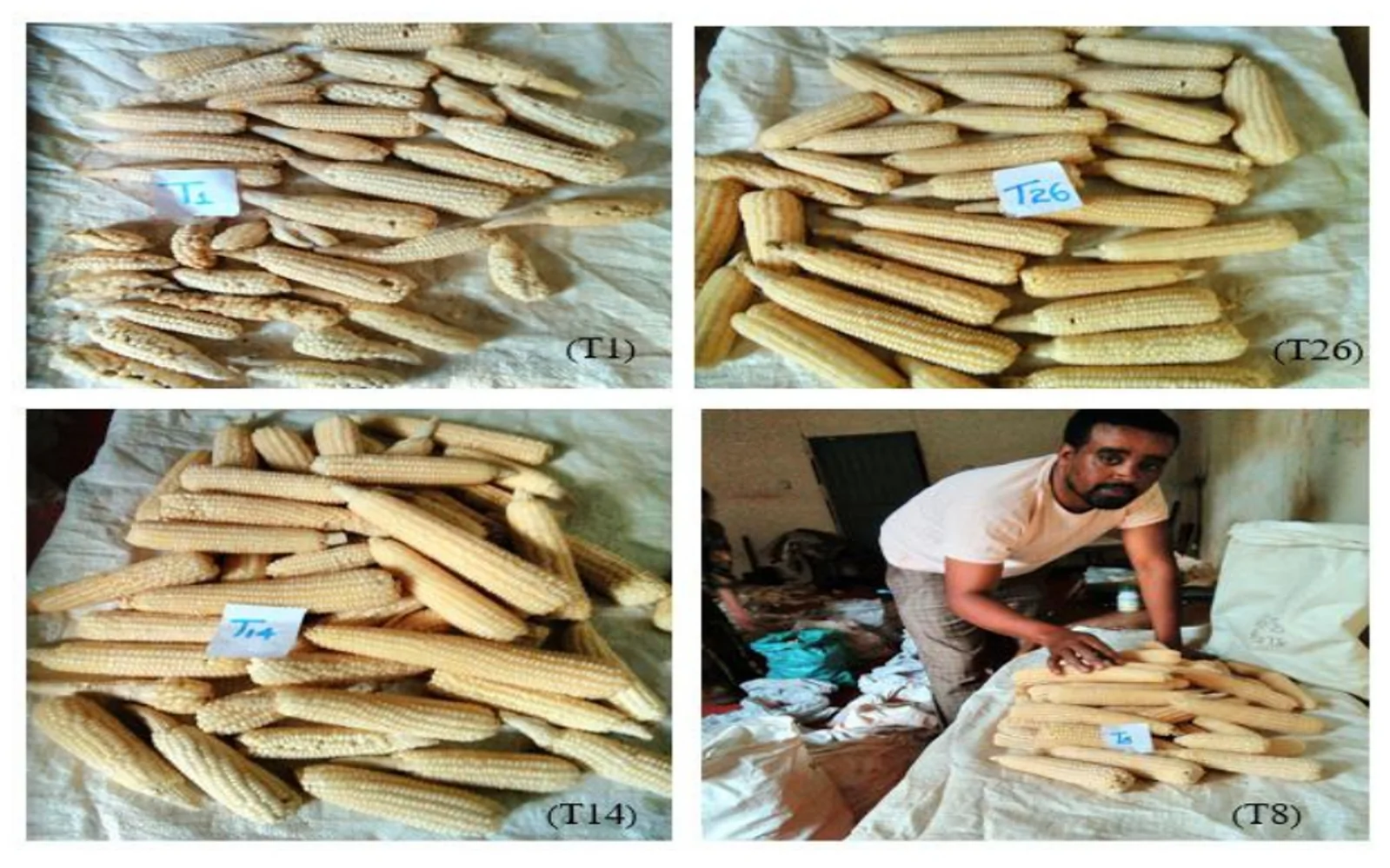
Sample maize cob with grains after de-husking but before threshing (20 February 2025)

Data were collected at 90% physiological maturity (including Days to 90% PM, NLPP, and PH) and after harvest (including yield-related traits and mean GY) (Fig. 7). Days to 90% physiological maturity (DPM) was recorded as the number of days after sowing until the formation of a black layer at the point where the kernel attaches to the cob. Plant height at harvest was measured in centimeters from the soil surface to the base of the tassel on ten randomly selected plants from the net plot area at physiological maturity. A 3 m steel ruler was used to measure PH following the method of [57]. Number of leaves per plant (NLPP) was determined from ten randomly selected plants in the middle three rows of each net plot, and the number of leaves per plant was counted to calculate the average.

#### Yield-related traits and yield

Yield and yield-related traits were recorded from ten tagged plants per replication. The mean of each trait was calculated for each replication at physiological and harvest maturity, and the data were expressed as mean values. Ear length (EL): The length of an ear, measured from the grain collar to the tip, was recorded on ten randomly selected representative plants from each experimental plot, and the mean value was calculated (Fig. 7). Number of rows per ear (NRPE): Ten plants were randomly selected from three central rows of each net plot, and counted the number of rows per ear and their average was worked out. The mean value was then calculated. Counting was done manually using a marker to reference and encircle the rows of the ear to minimize errors. Summing up the number of rows per ear and divided by ten to get the mean NRPE. Ear number per plant (ENPP): Ten randomly pre-tagged plants were taken from the net plot area, and then their ear was counted at harvest and the average was recorded.

Grains number per ear (GNPE): Ten plants ear were selected randomly from each net plot and number of grains were counted. Finally, the average was recorded. Thousand seed weight (TSW): Two samples of 1,000 grains were taken at random from each treatment using digital automatic seed counter (SLY-C, TPC311015) and then weighed on a digital balance, and the average was recorded. The 1000 grain weights was weighed after testing seed moisture content by using seed moisture tester instrument at the Ethiopia commodity exchange (ECX) office, Burie district branch. The final dry weight of 1000 grains were computed using 12.5% as market standard seed moisture content of maize. Biological yield (BY): At physiological maturity, plants from the net plot area (1.65 m × 2.5 m) were harvested and weighed after sun drying. It was measured with a weighing balance until a constant weight was achieved. The biological yield thus obtained in each plot was converted it into kg ha⁻¹ using Eq. 1 [50]:

$$\:Biological\:yield\:{(kg\:ha}^{-1})=(\frac{Biological\:yield\:\left(kg\right)}{net\:area\:harvested\:\left(1.65\:m\times\:2.5m\right)})\times\:\mathrm{10,000}\:\:\:\:\:\:\:\:\:\:\:\:\:\:(1)$$

After harvesting, maize cobs were manually de-husked, threshed, and winnowed on a plot-by-plot basis, with each treatment harvested separately. Grain moisture content was measured at harvest using a digital moisture meter and adjusted to the standard 12.5% level for maize in Ethiopia, following national recommendations [58, 59]. The threshed grains were air-dried and weighed, and the final grain yield was calculated and expressed in tonnes per hectare (t ha⁻¹) using Eq. 2 and Eq. 3 [50].

$$\:Grain\:yield\:{(kg\:ha}^{-1})=(\frac{Grain\:yield\:{(kg\:plot}^{-1})}{net\:area\:harvested\:\left(1.65\:m\times\:2.5m\right)})\times\:\mathrm{10,000}\:\:\:\:\:\:\:\:\:\:\:\:\:\:\:\:\:\:\:\:\:\:\:\:(2)$$

$$\:\mathrm{A}\mathrm{d}\mathrm{j}\mathrm{u}\mathrm{s}\mathrm{t}\mathrm{e}\mathrm{d}\:\mathrm{g}\mathrm{r}\mathrm{a}\mathrm{i}\mathrm{n}\:\mathrm{y}\mathrm{i}\mathrm{e}\mathrm{l}\mathrm{d}\:\left({kg\:ha}^{-1\:}\right)=\frac{Grain\:yield\:\:\left({kg\:ha}^{-1\:}\right)\:x\:\left(100-MC\right)}{(100-12.5)}\:\:\:\:\:\:\:\:\:\:\:\:\:\:\:\:\:\:\:\:\:\:\:\:\:\:\left(3\right)$$

Where, MC is measured moisture content in grain for each treatment and 12.5 is the designated moisture content (%) for correction of cereal crop standard MC.

### Economic and statistical analysis

#### Economic analysis

Partial budget analysis was conducted using mean adjusted grain and straw yields. For economic analysis the market price of maize GY, costs of inputs and labors are presented in Table 4. The product of maize yield for each treatment-year combination was used to calculate revenue (*R*). Benefits were calculated using the difference between revenues and expenditures. Net benefit and marginal rate of return (MRR) were computed based on [60] to determine economic viability. A 10% yield adjustment was applied to account for farmer-level realities, post-harvest losses and reflect real-world variability as demonstrated by [50, 61]. Since our study was conducted at research site, so 10% GY deduction from actual GY was conducted.

$$\:MRR\:\left(\%\right)=\frac{{NB}_{b\:}-{NB}_{a\:}}{{TVC}_{b\:}-{TVC}_{a\:}}\times\:100\:\:\:\:\:\:\:\:\:\:\:\:\:\:\:\:\:\:\:\:\:\:\:\:\:\:\:\:\:\:\:\:\:\:\:\:\:\:\:\:\:\:\:\:\:\:\:\:\:\:\:\:\:\:\:\:\:\:\:\:\:\:\:\:\:\:\:\:\:\:\:\:\:\:\:\:\:\:\:\:\:\:\:\left(4\right)$$

Abbreviations: NB_a_ is net benefit with the immediate lower total variable cost (TVC); NB_b_ is net benefit with the next higher TVC; TVC_a_ is the immediate lower TVC and TVC_b_ is the next highest TVC.

**Table 4:** Market price of inputs and labor cost used for economic analysis across two years value in ETB (1USD = 55.09 ETB)

Sn.	Items	Year		Mean (ETB)	Mean (USD)
		2023/24	2024/25		
1	Urea (100 kg⁻¹)	8589.98	4060.00	6324.99	114.81
2	NPSB (100 kg⁻¹)	10,000.00	4100.00	7200.00	130.70
3	Biochar (kg⁻¹)	10.20	0.125	5.16	0.093
4	Vermicompost (kg⁻¹)	3.80	0.46	2.13	0.04
5	Lime (100 kg⁻¹)	654.58	654.58	654.58	11.88
6	Seed (kg⁻¹)	88.00	56.00	72.00	1.31
7	Labor (person⁻¹day⁻¹)	200.00	250.00	225.00	4.08
8	Grain yield price (kg⁻¹)	27.22	28.45	27.83	0.50
9	Straw yield price (kg⁻¹)	4.30	4.56	4.40	0.08

#### Statistical analysis

Prior to data analysis, normality was tested using the Statistical Package for the Social Sciences (SPSS, version 27) [62]. The Shapiro-Wilk test was used to determine data normality. When the normality test was significant (*p* ≤ 0.05), data transformation was applied. Homogeneity of error variances was assessed using the F-test as outlined by [63]. Analysis of variance (ANOVA) was conducted using SAS software 9.4 [64] to evaluate treatment effects. When ANOVA indicated significant differences (*p* < 0.05), mean separation was performed using the least significant difference (LSD) test at the 5% significance level. Using (SPSS, version 27), a correlation analysis was also performed to define the association between plant growth parameters and yield and yield components as affected by rates of N/P_2_O_5_, BC and VC. Besides, graphical abstracts were prepared using OriginPro 2024 software.

## Results

### Correlation of maize growth, yield, and yield components

Correlation analysis of maize growth parameters revealed that all were highly and positively correlated with grain yield at Burie district (Table 5). The highest positive association and highly significant difference was recorded between TGW and GY (*r* = 0.809, *p* < 0.01), while the lowest positive association as well as highly significant difference was found between PH and GY (*r* = 0.464, *p* < 0.01). We found that 81% of the variation in GY was explained by TGW, while the remaining 19% might be influenced by environmental and management factors.

**Table 5:** Correlation coefficient matrix of the relationship between selected growth, yield component parameters, and maize yield at Burie district (2023-24 & 2024-25)

	DPM	NLPP	PH	EL	NEPP	NGPE	BY	NRPE	TGW	GY
DPM	--									
NLPP	0.495**	--								
PH	0.262^ns^	0.448*	--							
EL	0.642**	0.584**	0.418*	--						
NEPP	0.861**	0.719**	0.231^ns^	0.672**	--					
NGPE	0.678**	0.683**	0.459*	0.850**	0.759**	--				
BY	0.905**	0.581**	0.32^ns^	0.682**	0.888**	0.779**	--			
NRPE	0.236^ns^	0.591**	0.486*	0.644**	0.483*	0.670**	0.412*	--		
TSW	0.458*	0.690**	0.536**	0.759**	0.647**	0.846**	0.626**	0.806**	--	
GY	0.511**	0.759**	0.464*	0.719**	0.749**	0.759**	0.694**	0.737**	0.809**	--

*Abbreviations*: *DPM *days to 90% physiological maturity, *NLPP *number of leaves per plant, *PH* plant height at 90% PM, *EL* ear length, *NEPP* number of ears per plant, *NGPE* number of grains per ear, *BY *biological yield, *NRPE* number of rows per ear, *TSW* thousand grain weight, *GY* grain yield; **,*, and ns, highly significant (*p* < 0.01), significant (*p* < 0.05) and non-significant (*p* > 0.05) at 1 and 5% probability level

### Effect of inorganic N/P_2_O_5_, BC, VC and growing seasons on physiology and growth traits

#### Days to 90% physiological maturity

ANOVA across years revealed that the days to 90% PM of maize were highly significantly (*p* < 0.01) influenced by the three main effects, as well as the three-way interaction among N/P₂O₅, BC, and VC levels, in both individual years and when averaged over years (Tables 6 and 7; Fig. 9). The duration a crop takes to reach maturity plays a vital role in assessing its compatibility with particular environmental conditions and farming systems. In 2023, 2024, and on average across both years, maize took significantly longer to mature under the soil fertility management treatment involving in T27 as combined application of 10.04 t ha⁻¹ of VC, 240/138 kg ha⁻¹ of N/P₂O₅, and 8 t ha⁻¹ of BC, with recorded durations of 174.82, 174.65, and 174.73 days, respectively. This was closely followed by a similar treatment using 5.02 t ha⁻¹ of VC in 2023, 2024 and averaged mean days to 90% PM, which resulted in 170.72, 172.49, and 171.61 days to maturity, respectively (Table 6). The shortest days to 90% PM durations were observed in the control plot, with 142.1 days in 2023, 143.21 days in 2024, and an average of 142.11 days across both years (Table 6).

In 2023, maize reached 90% physiological maturity in 174.82 days under the treatment of 240/138 kg N/P₂O₅ ha⁻¹, 8 t BC ha⁻¹, and 10.04 t VC ha⁻¹. This duration was notably longer than that observed for other treatments, including 240/138 kg N/P₂O₅ ha⁻¹ alone (153.42 days), 120/69 kg N/P₂O₅ ha⁻¹ (148.78 days), 8 t BC ha⁻¹ alone (161.09 days), 4 t BC ha⁻¹ (154.78 days), 10.04 t VC ha⁻¹ (147.93 days), 5.05 t VC ha⁻¹ (146.99 days), and the control treatment (141.02 days).

Similarly, in 2024, maize reached 90% physiological maturity in 174.65 days under the combined application of 240/138 kg N/P₂O₅ ha⁻¹, 8 t BC ha⁻¹, and 10.04 t VC ha⁻¹, again recording the longest duration among all treatments. Earlier maturity was observed under 8 t BC ha⁻¹ alone (163.73 days), 4 t BC ha⁻¹ (156.71 days), 4 t BC ha⁻¹ (156.71 days), 240/138 kg N/P₂O₅ ha⁻¹ alone (154.64 days), 120/69 kg N/P₂O₅ ha⁻¹ (151.33 days), 10.04 t VC ha⁻¹ (150.56 days), 5.05 t VC ha⁻¹ (148.30 days), and the control treatment (143.21 days). This pattern reinforces the trend observed in 2023, highlighting the influence of integrated nutrient and organic inputs on extending the maturity period of maize.

Based on the average across both years, maize on the plot that received a combined application of 240/138 kg N/P₂O₅ ha⁻¹, 8 t BC ha⁻¹, and 10.04 t VC ha⁻¹ took the longest time to reach 90% physiological maturity, with an average of 174.73 days. This duration was considerably longer than that observed under other treatments: 240/138 kg N/P₂O₅ ha⁻¹ alone (154.55 days), 120/69 kg N/P₂O₅ ha⁻¹ (150.05 days), 8 t BC ha⁻¹ (162.41 days), 4 t BC ha⁻¹ (155.46 days), 10.04 t VC ha⁻¹ (149.24 days), and 5.02 t VC ha⁻¹ (147.64 days). The shortest maturity period was recorded in the control plot, with an average of just 142.11 days. These results further confirm the consistent trend across years, demonstrating that the integrated application of inorganic and organic amendments significantly delays maize maturity.

#### Plant height at maturity

At physiological maturity, analysis of variance revealed that PH was highly significantly affected (*p* < 0.01) by the main effects, as well as the two-way and three-way interactions of N/P₂O₅, BC, and VC during the 2023 and 2024 cropping seasons (Tables 6 and 7). Additionally, the interaction between BC and year (BC × year) had a significant effect (*p* < 0.05) on PH at maturity.

In 2023, the tallest maize plants (PH) were recorded with the application of 120/69 kg N/P₂O₅ ha⁻¹ + 4 t BC ha⁻¹ + 10.04 t VC ha⁻¹, reaching a significant maximum height of 317.76 cm. This was closely followed by the treatment of 240/138 kg N/P₂O₅ ha⁻¹ + 10.04 t VC ha⁻¹, which produced a PH of 315.88 cm. The PH observed under the treatment 240/138 kg N/P₂O₅ ha⁻¹ + 5.02 t VC ha⁻¹ (309.86 cm) was comparable to the previous two (Table 6). In contrast, the lowest average PH (218.18 cm) was recorded in the control treatment with no application of N/P₂O₅, BC, or VC.

Similarly, in 2024, the tallest plants were obtained from the application of 240/138 kg N/P₂O₅ ha⁻¹ + 5.02 t VC ha⁻¹ (313.7 cm), which was statistically comparable to 8 t BC ha⁻¹ alone (312.18 cm). These treatments were also not significantly different from the combination of 0 kg N/P₂O₅ ha⁻¹ + 8 t BC ha⁻¹ + 0 t VC ha⁻¹, which yielded a PH of 304.1 cm. The shortest plants were again observed in the control plots, with an average PH of 238.4 cm. Significantly greater mean PH in maize was achieved with the application of optimal inorganic N/P_2_O_5_ combined with 10.04 t VC ha⁻¹ (Table 6).

#### Number of leaves per plant

**Fig. 8:**
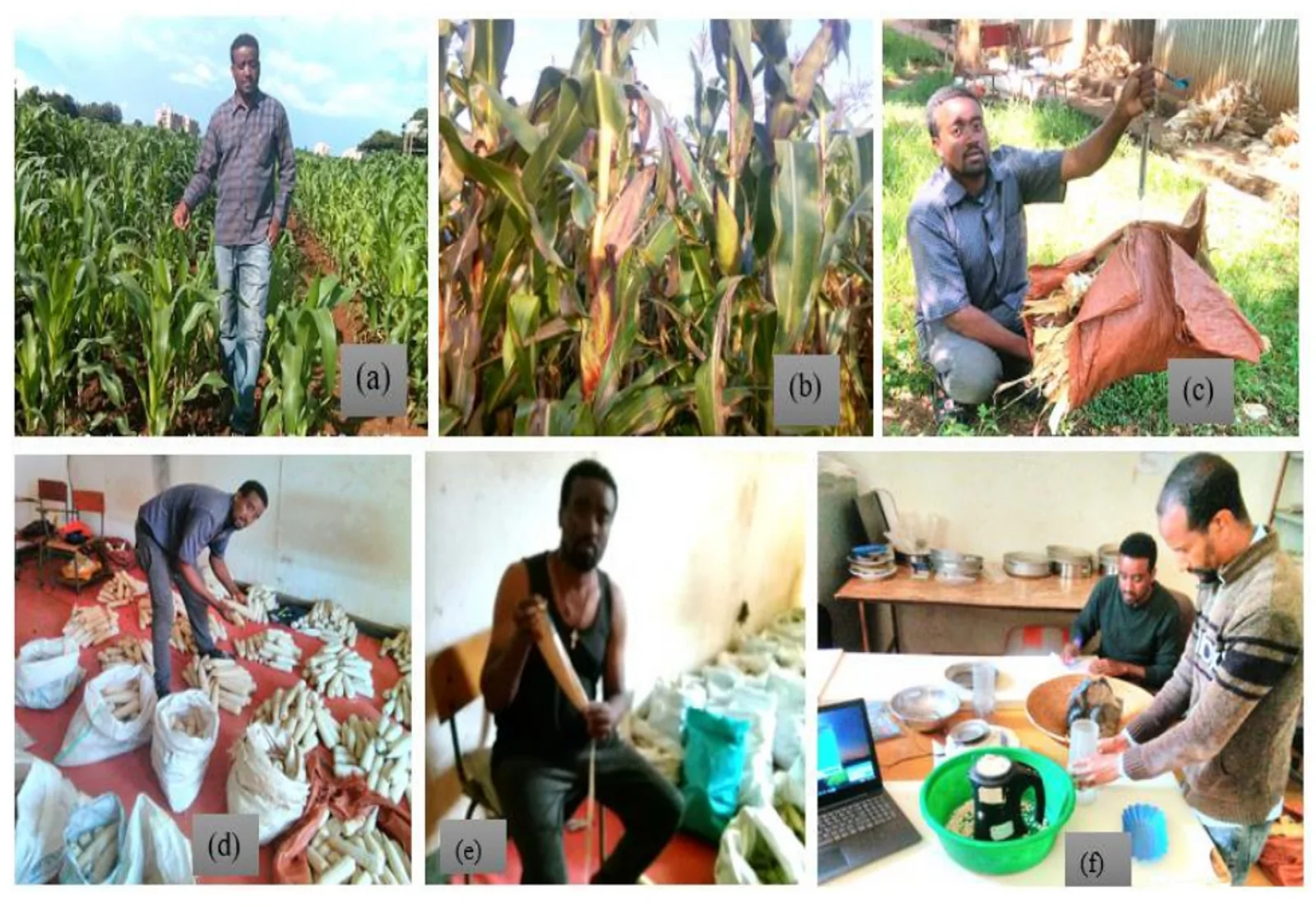
Illustration of physiological growth to grain yield moisture content measurement. (**a**) at knee height stage, (**b**) grain filling stage, (**c**) measuring biomass, (**d**) number of cobs per plot, (**e**), cob length measurement and (**f**) grain yield moisture content measurement (Photo by Habtamu Tadele, 2023-24 & 2024-25)

A significantly higher mean number of leaves per plant (NLPP) in maize was observed with the combined application of N/P₂O₅, BC, and the optimum rate of VC (Tables 6 and 7; Fig. 8). In 2023, the highest NLPP (17.07) was recorded from the interaction of 120/69 kg N/P₂O₅ ha⁻¹ + 8 t BC ha⁻¹ + 10.04 t VC ha⁻¹, while the lowest NLPP (13.04) occurred in the control treatment with no fertilizer applied. However, the difference was not statistically significant when compared to treatments T14 (120/69 kg N/P₂O₅ ha⁻¹ + 4 t BC ha⁻¹ + 5.02 t VC ha⁻¹), which recorded 16.86 leaves per plant, and T25 (240/138 kg N/P₂O₅ ha⁻¹ + 8 t BC ha⁻¹ + 0 t VC), with 16.88 leaves per plant. In 2024, the highest NLPP was recorded from the interaction of 240/138 kg N/P₂O₅ ha⁻¹ + 4 t BC ha⁻¹ + 0 t VC (18.99), followed by 240/138 kg N/P₂O₅ ha⁻¹ + 0 t BC ha⁻¹ + 5.02 t VC ha⁻¹ (18.19). The lowest NLPP (14.23) was observed in the control treatment without any fertilizer application.

Averaged over the two years, the highest NLPP (17.45) resulted from 120/69 kg N/P₂O₅ ha⁻¹ + 8 t BC ha⁻¹ + 10.04 t VC ha⁻¹, followed by 240/138 kg N/P₂O₅ ha⁻¹ + 4 t BC ha⁻¹ + 5.02 t VC ha⁻¹ (17.17) and 240/138 kg N/P₂O₅ ha⁻¹ + 0 t BC ha⁻¹ + 10.04 t VC ha⁻¹, (17.12). The lowest mean NLPP (13.63) was from the unfertilized control. This represents a 20.8% increase in leaf number due to the combined optimum use of inorganic nutrients, BC, and VC over the control.

**Table 6:** Three-way interaction effect of N/P_2_O_5_, BC and VC on mean days to 90% PHM, PH and NLPP over two seasons (2023-24 & 2024-25)

*N*/*P*_2_O_5_ (kg ha⁻¹)	BC (t ha⁻¹)	Days to 90% PM			Plant height at harvest (cm)			Number of leaves per plant		
		VC (t ha⁻¹)								
		0	5.02	10.04	0	5.02	10.04	0	5.02	10.04
0	0	142.11^u^	147.64^t^	149.24^s^	225.7^r^	248.1^p^	255.1^o^	13.63^p^	15.08^mn^	15.40^kl^
	4	155.46^n^	156.18^m^	157.51^l^	281.3^h^	258.9^mn^	260.7^lm^	15.20^lm^	14.89^no^	15.15^lmn^
	8	162.41^h^	163.28^g^	164.19^f^	303.9^c^	271.3^j^	268.3^k^	15.16^lm^	14.70^o^	15.92^hi^
120/69	0	150.05^r^	151.27^q^	153.28^p^	257.2^no^	270.7^jk^	295.9^e^	15.79ij	16.89^cd^	16.79^d^
	4	158.33^k^	158.68^k^	159.37^j^	289.5^fg^	280.4^h^	309.9^b^	15.54^jk^	16.33^ef^	16.15^f-h^
	8	165.20^e^	166.83^d^	167.48^d^	291.3^f^	274.3^i^	277.1^i^	15.28^lm^	16.13^fgh^	17.45^a^
240/138	0	154.55^o^	154.79^no^	155.32^n^	281.3^h^	299.3^d^	320.5^a^	16.03^ghi^	16.27^efg^	17.12^bc^
	4	159.95^j^	160.99^i^	161.91^h^	255.7^o^	286.6^g^	307.2^b^	15.96^hi^	17.17^b^	15.06^mn^
	8	169.45^c^	171.61^b^	174.73^a^	243.4^q^	263.0^l^	287.5^g^	16.74^d^	16.48^e^	15.92^hi^
Grand mean				158.95			276.48			15.86
F-test				< 0.0001			< 0.0001			< 0.0001
LSD (0.05)				0.225			0.98			0.085
R-Square				0.99			0.98			0.98
CV				0.34			1.18			1.35

There is no noticeable difference between means inside a column that is preceded by the same letter

**Table 7:** Mean square and significance test after combined analysis of variance for the effects of year, block, N/P_2_O_5_, BC, and VC, and their interactions on PM, PH, and NLPP at Burie district 2023-24 & 2024-25 (combined over two years)

Source of variation	DF	90% PM	PH	NLPP
Block	2	0.33^ns^	5.65^ns^	0.09^ns^
N/P_2_O_5_	2	671.17^**^	5997.37^**^	28.99^**^
BC	2	3593.30^**^	982.42^**^	5.99^**^
VC	2	109.05^**^	4430.54^**^	0.93^**^
Year	1	175.93^**^	1264.31^**^	59.83^**^
N/P_2_O_5_*BC	4	35.13^**^	6490.25^**^	3.30^**^
N/P_2_O_5_*VC	4	9.36^**^	3952.22^**^	1.20^**^
N/P_2_O_5_*Year	2	3.19^**^	215.40^**^	1.77^**^
BC*VC	4	16.68^**^	1575.99^**^	3.56^**^
BC*Year	2	9.60^**^	36.27^*^	5.14^**^
VC*Year	2	8.90^**^	1112.93^**^	2.72^**^
N/P_2_O_5_*BC*VC	8	11.32^**^	812.01^**^	2.07^**^
N/P_2_O_5_*BC*Year	4	1.22^**^	169.01^**^	5.11^**^
N/P_2_O_5_*VC*Year	4	3.91^**^	481.28^**^	1.47^**^
BC*VC*Year	4	10.35^**^	169.71^**^	1.84^**^
N/P_2_O_5_*BC*VC*Year	8	3.71^**^	516.6^**^	0.85^**^
Error		0.30	10.72	0.05

*Abbreviations*: *DF *Degree of freedom, *PM *days to 90% physiological maturity, *PH *plant height at harvest, *NLPP *number of leaves per plant

*P* value, ** and * significant at (1 and 5%) probability level respectively, *ns* non-significant at 1 and 5% probability level

### Effect of N/P_2_O_5_, BC, VC and growing seasons on yield and yield related traits

#### Ear length (EL)

The analysis of variance revealed that ear length was highly significantly influenced (*p* < 0.01) by the main effects of N/P₂O₅, BC, VC application rates, year, and their interactions in 2023, 2024, and across the averaged data (Tables 8 and 9; Fig. 8). However, the interaction between BC and year did not have a significant effect (*p* > 0.05) on EL (Table 9). Thus, EL is a key yield component in maize, as it substantially impacts grain production by determining both the number and size of grains. In 2023, maize EL was significantly increased by the combined application of N/P₂O₅, BC, and VC (Tables 8 and 9; Fig. 7). The longest EL, measuring 19.84 cm, was recorded when 240/138 kg N/P₂O₅ ha⁻¹ was applied together with 8 t BC ha⁻¹ and 10.04 t VC ha⁻¹. This was closely followed by the same N/P₂O₅ and BC rates combined with 5.02 t VC ha⁻¹ rate, which produced an EL of 19.68 cm. In contrast, the shortest EL (12.91 cm) was observed in the control.

In 2024, the longest EL (21.5 cm) was recorded when 120/69 kg N/P₂O₅ ha⁻¹ was combined with 4 t BC ha⁻¹ and 5.02 t VC ha⁻¹. This was statistically similar to the treatment of 240/138 kg N/P₂O₅ ha⁻¹ with 8 t BC ha⁻¹ and 5.02 t VC ha⁻¹, which produced an EL of 21.11 cm. The shortest EL (12.41 cm) was observed in the control plots. Regarding BC levels, in 2023, the highest EL (17.33 cm) was found in plots treated with 8 t BC ha⁻¹, while the lowest (15.9 cm) was recorded in plots without BC. Similarly, in 2024, the longest EL (17.62 cm) occurred in plots with 8 t BC ha⁻¹, and the shortest EL (16.31 cm) was in plots without BC. Averaged over the two years, the maximum EL (17.47 cm) was recorded in plots receiving 8 t BC ha⁻¹, compared to 16.1 cm in plots without BC. Across both growing seasons, the average EL was longest in 2024 (17.14 cm) and shortest in 2023 (16.66 cm).

Averaged over the years, the highest EL (20.39 cm) was achieved with the combined application of 120/69 kg N/P₂O₅ ha⁻¹, 8 t BC ha⁻¹, and 5.02 t VC ha⁻¹. This was comparable to the treatment of 240/138 kg N/P₂O₅ ha⁻¹, 8 t BC ha⁻¹, and 10.04 t VC ha⁻¹, which yielded an EL of 19.73 cm. The shortest EL (12.66 cm) was recorded in the untreated control.

#### Ear number per plant (ENPP)

Ear number was highly significantly (*p* < 0.01) influenced by the individual effects of N/P_2_O_5_, BC, and VC, as well as their interactions, across both years and the overall average (Table 9). Combined data showed significant increases (*p* < 0.01) under these treatments, with ear number improvements ranging from 17% to 49%.

In the 2023 cropping season, ENPP significantly increased (*p* < 0.01) under treatments combining 120/69 kg N/P₂O₅ ha⁻¹ + 8 t BC ha⁻¹ + 10.04 t VC ha⁻¹, sole 240/138 kg N/P₂O₅ ha⁻¹, sole 10.04 t VC ha⁻¹, and sole 8 t BC ha⁻¹ by 21.74% to 41.55% compared to the control. Treatment 24 (combined application) showed similar ear numbers to treatments T25, T22, and T23, and the lowest was 0.9 ears plant^-1^ in control. Similarly, in 2024, ENPP increased significantly (*p* < 0.01) under 240/138 kg N/P₂O₅ ha⁻¹ + 8 t BC ha⁻¹, sole 240/138 kg N/P₂O₅ ha⁻¹, sole 8 t BC ha⁻¹, and sole 10.04 t VC ha⁻¹ by up to 55.75% compared to control. The highest ENPP (1.74) was recorded with 240/138 kg N/P₂O₅ ha⁻¹ + 8 t BC ha⁻¹ (Table 8; Fig. 7).

#### Grain number per ear

Grain number per ear (GNPE) was highly significantly affected (*p* < 0.01) by N/P₂O₅, BC, VC, year, and their interactions (Tables 8 and 10). In 2023, the highest GNPE (592.9) was recorded with 240/138 kg N/P₂O₅ ha⁻¹ + 4 t BC ha⁻¹ + 10.04 t VC ha⁻¹, closely followed by 120/69 kg N/P₂O₅ ha⁻¹ + 4 t BC ha⁻¹ + 10.04 t VC ha⁻¹ (575.67), while the control had the lowest (242.31). In 2024, the maximum GNPE (722.7) occurred with 120/69 kg N/P₂O₅ ha⁻¹ + 4 t BC ha⁻¹ + 5.02 t VC ha⁻¹, followed by 240/138 kg N/P₂O₅ ha⁻¹ + 8 t BC ha⁻¹ + 5.02 t VC ha⁻¹ (703.55); control remained lowest (238.05). Averaged over both years, the highest GNPE (647.11) was under 120/69 kg N/P₂O₅ ha⁻¹ + 4 t BC ha⁻¹ + 5.02 t VC ha⁻¹.

**Table 8:** Three-way interaction effect of N/P_2_O_5_, BC and VC on mean EL, ENPP and NGPE over two seasons (2023-24 & 2024-25)

*N*/*P*_2_O_5_ (kg ha⁻¹)	BC (t ha⁻¹)	Ear length (cm)				Ear number per plant				Grain number per ear	
		VC (t ha⁻¹)									
		0	5.02	10.04	0	5.02	10.04	0	5.02		10.04
0	0	12.66^m^	14.28^l^	15.13^k^	0.83^s^	0.94^r^	1.00^q^	342.31^o^	503.17^m^		535.03^k^
	4	14.45^l^	14.09^l^	16.19^hi^	1.00^q^	1.22^kl^	1.23^jk^	489.37^n^	498.40^mn^		514.20^l^
	8	15.28^jk^	15.13^k^	15.82^ij^	1.14^m-o^	1.23^jk^	1.32^gh^	535.26^k^	541.26^k^		552.71^j^
120/69	0	16.01^i^	16.21^hi^	17.38^fg^	1.03^q^	1.08^p^	1.12^n-p^	519.07^l^	555.17^ij^		571.38^gh^
	4	16.77^gh^	19.03^c^	18.51^cd^	1.27^ij^	1.29^hi^	1.34^fg^	558.03^ij^	647.11^a^		609.27^de^
	8	16.71^h^	18.32^de^	18.31^de^	1.43^d^	1.46 cd	1.54^b^	571.47^gh^	613.61^cd^		630.25^b^
240/138	0	17.75^ef^	19.09^c^	16.44^hi^	1.11^op^	1.16^mn^	1.18^lm^	515.52^l^	580.23^g^		600.65^ef^
	4	17.76^ef^	18.75^cd^	18.50^cd^	1.36^ef^	1.39^e^	1.47^c^	564.17^hi^	568.04^h^		599.52^f^
	8	17.56^ef^	20.39^a^	19.73^b^	1.63^a^	1.36^ef^	1.38^e^	603.55^ef^	620.71^c^		607.38^d-f^
Grand mean			16.90			1.24			561.24		
F-test			0.0061			< 0.0001			< 0.0001		
LSD (0.05)			0.03			0.0136			3.17		
R-Square			0.96			0.97			0.98		
CV			2.76			3.33			1.46		

There is no noticeable difference between means inside a column that is preceded by the same letter

**Table 9:** Mean square results and p value for EL, ENPP and GNPE as affected by N/P_2_O_5_, BC and vermicompost rates and their interaction (combined over two year)

Source of variation	DF	EL	ENPP	GNPE
Block	2	0.15^ns^	0.001^ns^	6.04^ns^
N/P_2_O_5_	2	194.16**	0.83**	93744.93**
BC	2	27.24**	1.63**	33334.35**
VC	2	25.59**	0.10**	31655.87**
Year	1	8.93**	0.01**	150641.95**
N/P_2_O_5_*BC	4	1.97**	0.02**	3910.18**
N/P_2_O_5_*VC	4	6.92**	0.07**	1419.09**
N/P_2_O_5_*Year	2	7.73**	0.09**	9271.60**
BC*VC	4	0.85**	0.03**	2936.36**
BC*Year	2	0.63^ns^	0.04**	12113.03**
VC*Year	2	6.53**	0.01^ns^	3129.06**
N/P_2_O_5_*BC*VC	8	4.72**	0.03**	2212.97**
N/P_2_O_5_*BC*Year	4	1.77**	0.02**	3799.86**
N/P_2_O_5_*VC*Year	4	4.11**	0.001^ns^	1513.65**
BC*VC*Year	4	1.32**	0.001^ns^	2658.44**
N/P_2_O_5_*BC*VC*Year	8	2.17**	0.01**	2491.04**
Error	106	0.22	0.001	66.50

** and * Significant at (1 and 5%) probability level respectively, *ns* non-significant at 1 and 5% probability level, *DF *degree of freedom, *EL *ear length, *ENPP *ear number per plant and *GNPE *grain number per ear

#### Number of rows per ear (NRPE)

ANOVA for 2023 and 2024 showed that N/P₂O₅, BC, VC rates, and their three-way interaction significantly (*p* < 0.01) affected the NRPE (Tables 10 and 13). Combined analysis over years confirmed these effects were highly significant (*p* < 0.01), though interactions involving year were not significant (*p* > 0.05). In 2023, the highest NRPE (14.72) was recorded with 240/138 kg N/P₂O₅ ha⁻¹ + 0 t BC + 10.04 t VC ha⁻¹, statistically similar to several other treatments, while the control had the lowest (8.72). In 2024, the maximum NRPE (15.59) was observed under 120/69 kg N/P₂O₅ ha⁻¹ + 0 t BC + 5.02 t VC ha⁻¹, again significantly higher than the control (8.9). Averaged over both years, the highest NRPE (15.03) was from 240/138 kg N/P₂O₅ ha⁻¹ + 0 t BC + 10.04 t VC ha⁻¹, showing advantages over control and sole applications by 41.4%, 13.9%, 20.3%, and 15.0%, respectively.

#### Thousand grain weight (TGW)

ANOVA for 2023, 2024, and combined years showed that TGW was significantly (*p* < 0.01) influenced by N/P₂O₅, VC, BC, their interactions, and year (Tables 10 and 13). In 2023, the highest TGW (385.81 g) was recorded with 240/138 kg N/P₂O₅ ha⁻¹ + 10.04 t VC ha⁻¹, followed by treatments combining N/P₂O₅, BC, and VC. The control had the lowest TGW (244.9 g). In 2024, the highest TGW (511.25 g) was from 120/69 kg N/P₂O₅ ha⁻¹ + 4 t BC ha⁻¹ + 10.04 t VC ha⁻¹ (T15), with the control again lowest (224.73 g). Over both years, 120/69 kg N/P₂O₅ ha⁻¹ + 4 t BC ha⁻¹ + 10.04 t VC ha⁻¹ gave the highest average TGW (425.18 g), significantly outperforming the control by 44.8%, sole N/P₂O₅, BC, or VC applications (Table 10).

**Table 10:** Three way-interaction effect of N/P_2_O_5_, BC and VC on mean NRPE, TGW and BY over two seasons (2023-24 & 2024-25)

*N*/*P*_2_O_5_ (kg ha⁻¹)	BC (t ha⁻¹)	Number of rows ear⁻¹				TGW (g)		
		V (t ha⁻¹)						
		0	5.02	10.04	0		5.02	10.04
0	0	8.81^q^	11.37^p^	12.77^k−m^	234.57^p^		302.61^n^	308.33^l−n^
	4	11.82^op^	13.03^jk^	13.39^h−j^	311.51^lm^		292.70^o^	357.09^fg^
	8	11.99^no^	12.53^lm^	12.44^mn^	285.03^o^		304.65^mn^	322.20^jk^
120/69	0	12.87^k−m^	14.96^a^	13.80^e−h^	314.40^kl^		346.10^h^	382.19c^d^
	4	13.97^c−g^	14.28^cd^	14.06^c−e^	377.95^de^		400.40^b^	425.18^a^
	8	12.45^mn^	12.96^j−l^	13.58^f−h^	336.89^i^		351.01^gh^	407.74^b^
240/138	0	12.94^jkl^	14.03^c−f^	15.03^a^	327.86^j^		360.51^f^	407.77^b^
	4	13.71^e−h^	14.43^bc^	14.90^ab^	356.98^fg^		344.16^hi^	399.86^b^
	8	13.85^d−h^	13.53^g−i^	13.11^i−k^	388.37^c^		360.76^f^	373.57^e^
Pooled mean			13.29				347.42	
F-test			< 0.0001				< 0.0001	
LSD (0.05)			0.15				2.72	
R-Square			0.93				0.99	
CV			2.89				1.94	

There is no noticeable difference between means inside a column that is preceded by the same letter

#### Biological yield

Analysis of variance for 2023, 2024, and combined data showed that BY was highly significantly (*p* < 0.01) influenced by N/P₂O₅, BC, VC, year, and their interactions (Tables 11 and 12, and Table 13). In 2023, the highest BY (29.79 t ha⁻¹) was from 240/138 kg N/P₂O₅ ha⁻¹ + 8 t BC ha⁻¹ + 0 t VC, followed by 240/138 kg N/P₂O₅ ha⁻¹ + 8 t BC ha⁻¹ + 5.02 t VC ha⁻¹ (28.05 t ha⁻¹). The control had the lowest BY (8.11 t ha⁻¹), showing a difference of about 21.68 t ha⁻¹ due to nutrient synergy. In 2024, the top BY (29.78 t ha⁻¹) came from 120/69 kg N/P₂O₅ ha⁻¹ + 4 t BC ha⁻¹ + 5.02 t VC ha⁻¹, while the control again had the lowest (7.34 t ha⁻¹), differing by 22.44 t ha⁻¹, likely due to nutrient depletion. Averaged over yea data showed maximum BY (27.87 t ha⁻¹) with 240/138 kg N/P₂O₅ ha⁻¹ + 8 t BC ha⁻¹ + 0 t VC, statistically similar to 120/69 kg N/P₂O₅ ha⁻¹ + 8 t BC ha⁻¹ + 10.04 t VC ha⁻¹ (26.53 t ha⁻¹) and 240/138 kg N/P₂O₅ ha⁻¹ + 8 t BC ha⁻¹ + 10.04 t VC ha⁻¹ (26.66 t ha⁻¹). This treatment outperformed the control, sole maximum inorganic nutrients, BC, and VC by 72.3%, 19.8%, 13.6%, and 36.9%, respectively.

#### Grain yield (GY)

**Fig. 9:**
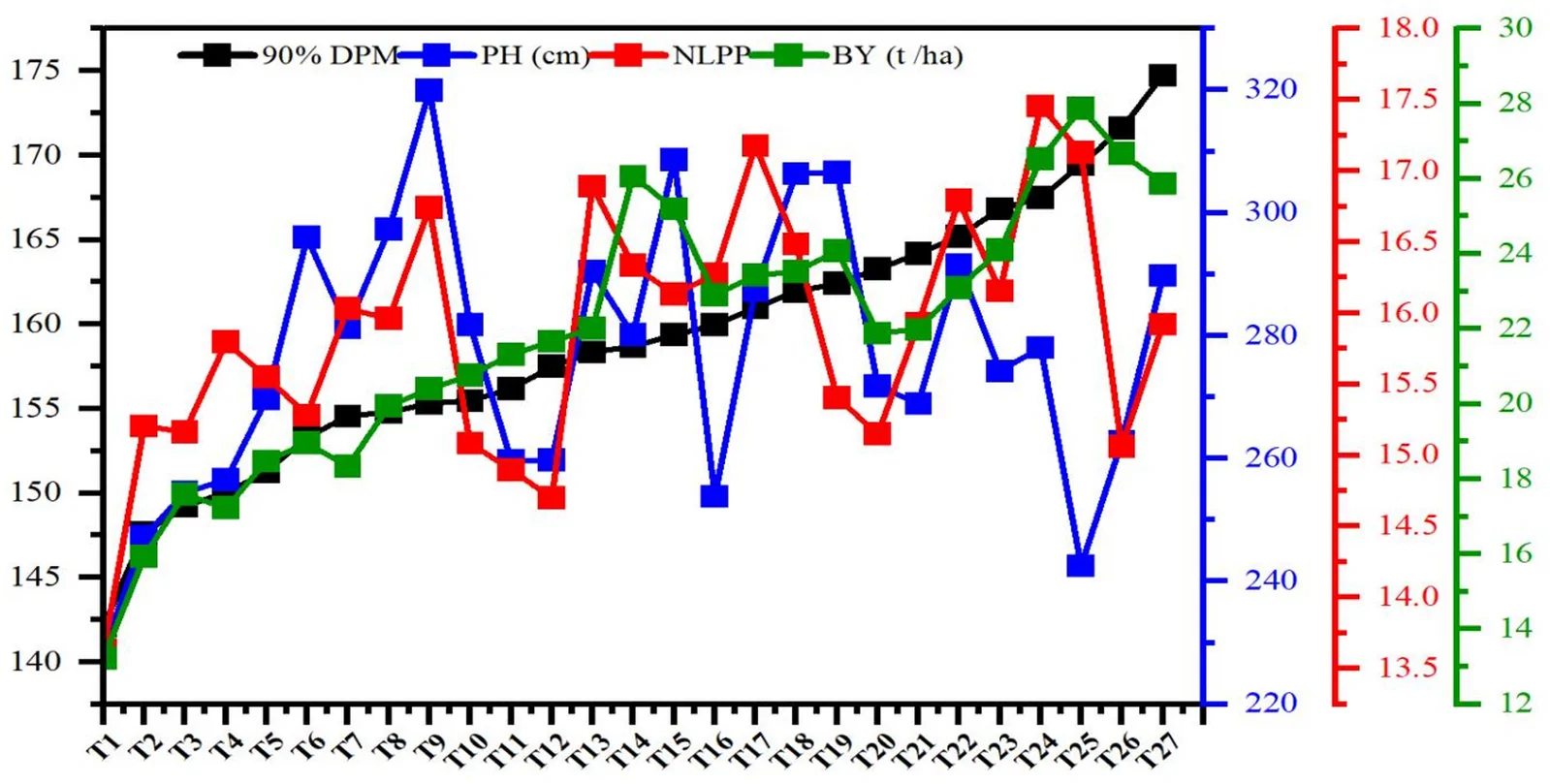
Mean days to 90% physiological maturity, plant height, number of leaves per plant and biological yield in 2023-24 & 2024-25

Maize GY was significantly affected by the interaction of N/P₂O₅, BC, and VC (Tables 11, 12 and 13). As shown in Table 11, the mean maximum GY was found a magnitude of decrease in yield in T24 > T14 > T17 > T16 > T18 > T15 > T1 (the control). In T24, T14 and T17 GY was increased by 63.73%, 63.6% and 62.5%, compared to the control during the experiment. The highest grain yield (GY) of 12.13 t ha⁻¹ was recorded in T24, followed closely by T14, which produced 12.09 t ha⁻¹, while the control had the lowest yield of 4.40 t ha⁻¹. However, the highest economic yield was obtained from T14. Sole applications of N/P₂O₅, BC, or VC resulted in lower GY. A half dose of inorganic N-P nutrients combined with moderate BC and VC (T14) produced yields 29.82% higher than the full recommended inorganic dose alone (T7). This might be due to their combined application improves soil fertility and crop growth more effectively than using each input individually. Treatments, T14 and T26 also showed notable gains, with T14 yielding 12.09 t ha⁻¹ (Table 11; Fig. 9).

**Table 11:** Interaction effect of different rates of inorganic N/P_2_O_5_ nutrients, BC and VC on BY and GY in 2023-24 & 2024-25

Treatment	*N*/*P*_2_O_5_ (kg ha⁻¹)	BC (t ha⁻¹)	VC (t ha⁻¹)	Biological yield (t ha⁻¹)				GY (t ha⁻¹)		
				2023	2024	COY	2023		2024	COY
1	0	0	0	15.11^z^	11.34^x^	13.22^t^	5.13^v^		3.67^s^	4.40^p^
2	0	0	5.02	16.71^y^	15.15^w^	15.93^s^	6.97^u^		6.22^r^	6.59^o^
3	0	0	10.04	17.61^x^	17.55^t^	17.58^q^	7.38^t^		6.95^qr^	7.16^n^
4	120/69	0	0	18.28^w^	16.18^v^	17.23^r^	8.17^pq^		7.01^qr^	7.59^mn^
5	120/69	0	5.02	18.84^v^	18.10^s^	18.47^p^	8.37^op^		8.77^j-m^	8.57^j^
6	120/69	0	10.04	19.32^u^	18.59^r^	18.95^o^	8.76^mn^		8.24^m-p^	8.50^jk^
7	240/138	0	0	19.75^tu^	16.93^u^	18.34^p^	9.36^ij^		7.74^o-q^	8.55^j^
8	240/138	0	5.02	20.16st	19.74^p^	19.94^n^	9.66^gh^		10.06^e-h^	9.86^gh^
9	240/138	0	10.04	20.53^rs^	20.26^o^	20.40^m^	9.82^j^		10.61^c-f^	10.21^fg^
10	0	4	0	20.89^qr^	20.64^no^	20.76^l^	7.88^rs^		7.97^n-p^	7.93^lm^
11	0	4	5.02	21.23^pq^	21.38^lm^	21.31^k^	8.08^qr^		8.393^l-o^	8.24^j-l^
12	0	4	10.04	21.58^op^	21.75^kl^	21.66^j^	10.34^f^		9.81^f-i^	10.07^fg^
13	120/69	4	0	21.90^no^	22.13^jk^	22.01^h^	10.44^f^		10.29^d-g^	10.37^ef^
14	120/69	4	5.02	22.34^mn^	29.78^a^	26.06^c^	11.49^c^		12.68^a^	12.09^a^
15	120/69	4	10.04	22.46^lm^	27.94^b^	25.19^d^	11.10^d^		11.21^bc^	11.15^cd^
16	240/138	4	0	22.89^kl^	22.91^i^	22.90^h^	11.59^c^		11.42^bc^	11.50^bc^
17	240/138	4	5.02	23.23^jk^	23.61^h^	23.42^fg^	12.19^b^		11.25^bc^	11.72^ab^
18	240/138	4	10.04	23.59^ij^	23.46^h^	23.52^f^	10.87^e^		11.61^b^	11.24^c^
19	0	8	0	23.94^hi^	24.21^g^	24.08^e^	7.69^s^		7.49^pq^	7.59^mn^
20	0	8	5.02	24.33^gh^	19.43^pq^	21.88^ij^	7.95^r^		8.19^n-p^	8.07^kl^
21	0	8	10.04	24.74^g^	19.21^q^	21.97^ij^	9.51^hi^		9.44^g-k^	9.47^hi^
22	120/69	8	0	25.19^f^	21.01^mn^	23.10^gh^	9.21^jk^		9.08^i-m^	9.14^i^
23	120/69	8	5.02	25.69^e^	22.51^ij^	24.10^e^	10.67^e^		10.88^b-e^	10.77^de^
24	120/69	8	10.04	26.28^d^	26.77^c^	26.53^b^	13.15^a^		11.12^b-d^	12.13^a^
25	240/138	8	0	29.79^a^	25.95^d^	27.87^a^	9.05^kl^		9.62^g-j^	9.34^i^
26	240/138	8	5.02	28.05^b^	25.28^e^	26.66^b^	8.86^lm^		9.26^h-l^	9.06^i^
27	240/138	8	10.04	27.01^c^	24.72^f^	25.86^c^	8.56^no^		8.58^k-o^	8.57^j^
Pooled mean				22.278	21.35	21.82	9.34		9.17	9.258
LSD (5%)				0.1478**	0.1412**	0.1002**	0.0698**		0.295**	0.1518**
R^2^				0.996	0.997	0.997	0.996		0.9525	0.97
CV (%)				1.215	1.211	1.203	1.37		5.889	4.296
Year										
Year 1 (2023)										9.34^a^
Year 2 (2024)										9.17^b^
Mean										9.258
LSD (0.05)										0.5863**
CV (%)										20.40

*Abbreviations*: *LSD *least significant difference, *BC *maize cob biochar *CV *coefficient of variation (%)

** Significant at 1 probability level, *DF* Degree of freedom

Means in the column within a parameter followed by the same letter(s) is not significantly different at *p* = 0.05

**Table 12:** The main effect of N/P_2_O_5_ nutrients, maize cob Biochar and vermicompost on BY and GY in 2023-24 & 2024-25

Treatments	Biological yield (t ha⁻¹)			Grain yield (t ha⁻¹)		
	2023	2024	COY	2023	2024	COY
N/P_2_O_5_ (kg ha⁻¹)						
0	20.685^c^	18.96^b^	19.82^c^	7.88^c^	7.57^b^	7.72^b^
120/69	22.258^b^	22.55^a^	22.40^b^	10.15^a^	9.92^a^	10.03^a^
240/138	23.891^a^	22.54^a^	23.21^a^	9.99^a^	10.02^a^	10.00^a^
LSD (0.05)	1794.2**	2.06**	1.79**	0.781**	0.9115**	0.8135**
CV (%)	14.87	17.86	15.15	15.42	18.33	16.21
BC (t ha⁻¹)						
0	18.483^b^	17.09^c^	17.78^c^	8.18^c^	7.7^c^	7.94^c^
4	22.236^a^	23.73^a^	22.98^b^	10.44^a^	10.51^a^	10.48^a^
8	26.115^b^	23.23^b^	24.67^a^	9.41^b^	9.29^b^	9.35^b^
LSD (0.05)	845.52**	1.52**	1.08	0.8232**	0.9027**	0.8307**
CV (%)	7.001	13.13	9.17	16.25	18.16	16.55
VC (t ha⁻¹)						
0	21.974^c^	20.14^a^	21.06^a^	8.72^b^	8.25^b^	8.49^b^
5.02	22.289^b^	21.66^a^	21.97^a^	9.36^ab^	9.52^a^	9.44a^b^
10.04	22.571^a^	22.25^a^	22.41^a^	9.95^a^	9.73^a^	9.83^a^
LSD (0.05)	1933.2**	2.21^ns^	1.94^ns^	0.932*	1.048*	0.9635*
CV (%)	16.008	19.16	16.41	18.39	21.08	19.2
Year						
Year 1 (2023)			22.28^a^			9.34^a^
Year 2 (2024)			21.36^a^			9.17^b^
Mean			21.82			9.258
LSD (0.05)			1.186^ns^			0.5863**
CV (%)			17.52			20.40

*LSD *least significant difference, *CV *coefficient of variation (%), *COY *combined over year, *SE *Standard error of a mean

Means in the same column followed by the same letter(s) are not statistically significant different at 5% probability level by LSD

**Table 13:** Mean square values for the effects of year, block, N/P_2_O_5_, BC and VC, and their interactions on EL, ENPP, GNPE, TGW and BY at Burie district (2023-24 & 2024-25)

Source of variation	DF	EL	ENPP	GNPE	NRPE	TGW	BY	GY
Block	2	0.15^ns^	0.001^ns^	6.04^ns^	0.01^ns^	66.7^9ns^	0.07^ns^	0.21^ns^
N/P_2_O_5_	2	194.16^**^	0.83^**^	93744.93^**^	45.22^**^	83354.19^**^	169.41^**^	94.81**
BC	2	27.24^**^	1.63^**^	33334.35^**^	8.97^**^	13213.97^**^	695.60^**^	87.44**
VC	2	25.59^**^	0.10^**^	31655.87^**^	13.73^**^	35848.20^**^	25.75^**^	25.91**
Year	1	8.93^**^	0.0^**^	150641.95^**^	1.70^**^	74558.91^**^	34.42^**^	1.22**
N/P_2_O_5_*BC	4	1.97^**^	0.02^**^	3910.18^**^	2.97^**^	3689.22^**^	14.49^**^	13.80**
N/P_2_O_5_*VC	4	6.92^**^	0.07^**^	1419.09^**^	1.02^**^	1257.23^**^	8.82^**^	5.87**
N/P_2_O_5_*Year	2	7.73^**^	0.09^**^	9271.60^**^	0.001^ns^	20101.93^**^	15.62^**^	0.41^ns^
BC*VC	4	0.85^**^	0.03^**^	2936.36^**^	2.30^**^	3793.66^**^	14.04^**^	1.59**
BC*Year	2	0.63^ns^	0.04^**^	12113.03^**^	1.21^**^	10496.34^**^	66.88^**^	1.08**
VC*Year	2	6.53^**^	0.01^*^	3129.06^**^	0.15^ns^	15103.81^**^	8.58^**^	1.37**
N/P_2_O_5_*BC*VC	8	4.72^**^	0.03^**^	2212.97^**^	2.12^**^	1857.74^**^	8.13^**^	2.85**
N/P_2_O_5_*BC*Y	4	1.77^**^	0.02^**^	3799.86^**^	0.66^**^	11694.08^**^	7.22^**^	0.83**
N/P_2_O_5_*VC*Y	4	4.11^**^	0.001^**^	1513.65^**^	0.94^**^	1574.90^**^	6.64^**^	1.16**
BC*VC*Year	4	1.32^**^	0.001^ns^	2658.44^**^	0.45^*^	2361.06^**^	4.81^**^	1.41**
N/P_2_O_5_*BC*VC*Y	8	2.17^**^	0.01^ns^	2491.04	0.94^**^	1862.59^**^	5.98^**^	0.46**
Error		0.22	0.001	66.50	0.15	45.49	0.07	0.16

*Abbreviations*: *DF *degree of freedom, *Y *year, *EL *ear length, *EN *ear number per plant, *GNPE* grain number pear ear, *NRPE *number of rows per ear, *TGW *1000 grain weight, *BY *biological yield

** and * significant at (1 and 5%) probability level respectively, *ns *non-significant at 1 and 5% probability level

### Partial budget analysis

Partial budget analysis results are presented in Table 14. The study revealed that sole applications of high N/P₂O₅ nutrient rates (e.g., 240/138 kg ha⁻¹, 10.04 t VC ha⁻¹and 8 t BC ha⁻¹) were not economically viable despite higher yields, due to their dominance and cost. For instance, 240/138 kg N/P₂O₅ ha⁻¹ yielded a net benefit of 193,957.95 ETB ha⁻¹ but was economically dominated. In contrast, integrated treatments showed significantly better profitability. Net benefit was higher in the order of T14, T16, T13, T17, T24, T15, T12, T23 and T10. However, treatments gave a marginal rate of return above the minimum acceptable rate of return (100%) were T10, T13 and T14. The most profitable treatment was 120/69 kg N/P₂O₅ ha⁻¹ + 5.02 t VC ha⁻¹ + 4 t BC ha⁻¹ (T14), which resulted in a net benefit of 289,124 ETB ha⁻¹ and a marginal rate of return (MRR) of 149.07%. This indicates a return of 1 ETB and 49 cents for every 1 ETB invested. Hence, T14 was followed by T13 and T10, all surpassing the acceptable MRR threshold of 100%, confirming their profitability under local price conditions. Sole application of inorganic N/P_2_O_5_, BC and VC were thus not very profitable.

**Table 14:** Partial budget analysis of maize yield for the determination of economically profitable level of N/P_2_O_5_ nutrients, BC and VC rate combined over years (2023-24 & 2024-25)

Treatments	*N*/*P*_2_O_5_ (kg ha⁻¹)	Biochar (t ha⁻¹)	VC (t ha⁻¹)	AGY GY (kg ha⁻¹)	ASY SY (kg ha⁻¹)	GB (ETB ha⁻¹)	Mean TVC (ETB ha⁻¹)	NB (ETB ha⁻¹)	MRR (%)
T1	0	0	0	4170.68	8211.00	156473.24	22694.20	128546.20	---
T10	0	4	0	7570.42	11660.83	262077.38	44094.20	216834.10	413
T2	0	0	0.50	6279.72	9074.97	215815.68	46225.05	167288.50	-2325.16**D**
T4	120/69	0	0	7219.90	9447.00	241732.76	48549.72	192486.65	-546.46**D**
T19	0	8	0	7242.75	14221.07	269492.52	65444.20	197914.70	-88.66 **D**
T11	0	4	0.50	7870.17	11884.67	269138.39	67625.05	202630.25	-60.36**D**
T3	0	0	10.04	6835.23	10034.25	239638.82	69755.90	163503.90	-207.8**D**
T13	120/69	4	0	9893.83	11437.23	326018.82	69949.72	254047.30	143.93
T5	120/69	0	5.04	8192.47	9656.63	270944.29	72080.57	197037.80	-2675.43**D**
T7	240/138	0	0	8125.35	9725.52	265101.37	73205.16	193957.95	-1845.81**D**
T20	0	8	5.04	7708.90	11540.57	268727.13	88975.05	175104.95	-413.93**D**
T12	0	4	10.04	9605.55	11430.43	294839.88	91155.90	224789.70	-137.97**D**
T22	120/69	8	0	8726.05	12025.77	293950.73	91299.72	202984.45	-239.17**D**
T14	120/69	4	5.02	11566.85	14102.17	376565.94	93480.57	289124.10	149.067
T16	240/138	4	0	10978.55	11437.55	349927.50	94605.16	259307.25	-2651.35**D**
T6	120/69	0	10.04	8105.05	10277.20	269576.53	95611.42	173719.90	**-**11468.6**D**
T8	240/138	0	5.02	9424.77	10038.48	302354.54	96736.01	208110.55	-2488.36**D**
T17	240/138	4	5.02	11169.63	11912.87	353117.83	110153.01	251050.60	-228.36**D**
T21	0	8	10.04	9044.20	10830.47	295056.13	112505.90	185156.65	-546.47**D**
T23	120/69	8	5.02	10290.42	11969.65	339147.26	114830.57	222452.15	-312.28**D**
T25	240/138	8	0	8927.22	14624.85	312246.75	115955.16	195766.00	-415.39**D**
T15	120/69	4	10.04	10654.92	14007.10	354327.23	117011.42	239743.25	-209.86**D**
T9	240/138	0	10.04	9771.08	10063.78	314128.63	120266.86	194325.90	-353.91**D**
T24	120/69	8	10.04	11536.00	14064.73	368168.20	138361.42	242439.75	-104.02**D**
T26	240/138	8	5.02	8656.50	14669.63	305812.28	139486.01	164878.15	-270.07**D**
T18	240/138	4	10.04	10747.9	11706.55	348612.7	141666.85	207248.25	-169.92**D**
T27	240/138	8	10.04	8182.93	14648.36	294412.6	163016.85	128118.1	-231.54**D**

*Abbreviations*: *AGY *adjusted grain yield, *ASY *adjusted straw yield, *GB *gross benefit, *TVC *total variable cost, *D *Dominated treatments, *MRR* marginal rate of return, *NB *net benefit

Maize yield was calculated on the selling price of maize at 27.22 ETB kg⁻¹) (2024) and 28.45 ETB (2025) cropping season (mean 27.83 ETB kg⁻¹ of maize)

## Discussion

### United use of inorganic N/P_2_O_5_ nutrients, BC and VC on associations of maize parameters

A comparison of the correlation coefficients revealed that TGW had a higher correlation coefficient (*r* = 0*.809*∗∗) than the other yield components, followed by NGPE (*r* = 0.759∗∗), NLPP (*r* = 0.759∗∗), NRPE (*r* = 0.737∗∗), NEPE (*r* = 0.749∗∗), EL(*r* = 0.719∗∗), BY (*r* = 0.694∗∗), PH (*r* = 0.464∗∗), and the days to 90% physiological maturity (*r* = 0.511∗∗). This showed associations with grain yield with correlation coefficients of comparatively good extents as compared to growth and physiological traits. Similarly [50, 59, 65], discovered that maize grain yields were positively and significantly associated with yield components.

### Influence of organic BC, VC, and inorganic N/P_2_O_5_ nutrients on physiology and growth traits

#### Days to 90% DPM

The response of crops to organic and inorganic nutrient sources to plant crops is determined by various factors, including rainfall, temperature, time of application, initial soil fertility status, growth stage, nutrient contents of inputs, and fertilizer use efficiency of the crop. This is important because environmental stress should not coincide with the sensitive growth stages. The results of our study indicate that the combined application of VC, BC, and N/P₂O₅ significantly influenced maize physiological maturity. The substantial differences in days to maturity between the control and the treated plots suggest a positive impact of these inputs on maize growth and development. Our findings indicate that the combined use of higher rates of inorganic and organic fertilizers prolongs maize growth up to 90% physiological maturity. This delay in maturity is primarily attributed to improved nutrient availability, especially nitrogen, which supports an extended vegetative growth phase and lengthens the growing period. The synergistic interaction between blended fertilizers (NPSB), BC, and VC enhances nutrient supply and soil health.

In particular, cob BC improves soil quality and facilitates more efficient nutrient uptake, encouraging a longer vegetative stage. Vermicompost enriches the soil with a broad array of essential nutrients, further enhancing fertility and contributing to the delayed phenological progression. Overall, the extended vegetative period reflects maize’s response to better nutrient and soil conditions, resulting in prolonged growth before reaching maturity.

Our findings align with previous studies showing that combined applications of lime, vermicompost, and mineral fertilizers significantly prolong crop maturity. In line with this [66], reported a 17-day extension in maize maturity with high rates of lime, vermicompost, and phosphorus fertilizer in Ethiopia. Similarly [67, 68], found increased maize maturity when combining organic amendments with recommended fertilizer doses. Micronutrients like boron and sulfur enhance nitrogen use efficiency and root development, contributing to this effect [69, 70]. In line with this [50] and [71], also demonstrated that integrated soil fertility management delays maize maturity compared to controls. Likewise [72], observed prolonged maturity with combined inorganic and organic inputs, highlighting the role of balanced nutrition in extending maize growth cycles.

#### Plant height at 90% DPM

Based on averaged across two years data, the tallest PH (320.5 cm) was recorded under the interaction of 240/138 kg N/P₂O₅ ha⁻¹ + 0 t BC ha⁻¹ + 10.04 t VC ha⁻¹, and the shortest PH (225.7 cm) was observed in the control (0.63 t CaCO_3_ ha⁻¹). These results demonstrate that combining organic fertilizers with inorganic nutrients enhances maize PH compared to unfertilized plots. The significant increase in PH is largely due to the combined and individual effects of nutrients, with nitrogen playing a key role in yield development. This was especially apparent in plants treated with the highest levels of N/P₂O₅, BC, and VC.

Our results align with numerous studies demonstrating that combined applications of organic and inorganic nutrients significantly increase plant height. Nitrogen plays a key role by enhancing chlorophyll synthesis and vegetative growth, while phosphorus and sulfur support rapid cell division and photosynthesis [73–75]. Earlier studies have reported similar findings: the integration of compost, VC, BC, and mineral fertilizers consistently improves maize and other cereal crop growth compared to unfertilized controls [76–80]. These studies collectively highlight that nutrient synergy from organic and inorganic sources promotes stronger vegetative development and greater final plant height.

#### Number of leaves per plant at 90% PM

Overall, combining organic and inorganic nutrients increased maize leaf number compared to unfertilized plants. The higher NLPP at increased rates of N/P₂O₅, BC, and VC likely results from reduced nutrient competition and the synergistic effects of organic and blended NPSB fertilizers on plant growth. More leaves enhance photosynthetic capacity, promoting better growth, can support better grain filling and yield potential. The other probable reasons might be, the combined application of nitrogen and phosphorus with BC and VC improves nitrogen use efficiency, supporting vigorous vegetative development. Poor nutrient management limits leaf production due to insufficient nutrient availability. The improved soil physicochemical properties and nutrient status from these amendments stimulate plant growth and leaf formation, particularly in acidic soils. The increase in NLPP reflects the positive effect of integrated nutrient management on vegetative growth and inter-nodal extension driven mainly by nitrogen availability.

Our findings align with [1], who reported the highest leaf number with combined N/P/S fertilizer and compost. Similarly [81], found more leaves per plant with higher soil amendments. Likewise [82], observed maximum green leaves under poultry manure and nitrogen, while [83] noted increased leaves with combined inorganic fertilizer and FYM. Besides [80], also showed that lime plus organic and inorganic fertilizers improved barley tiller numbers in acidic soils.

### Combined application of N/P_2_O_5_, BC and VC influence on yield and yield related traits

#### Ear length

The increased EL observed with the combined application of 120/69 kg N/P₂O₅ ha⁻¹, 4 t BC ha⁻¹, and 5.02 t VC ha⁻¹ is likely due to improved nutrient availability, which supports greater biomass production and more efficient photosynthetic allocation, resulting in longer ears. Our results agree with [84], who found that 100% NPK combined with vermicompost produced the longest cobs, while controls had the shortest. Similarly [85], reported increased ear length with higher nitrogen levels. Likewise [86], observed the longest ears with vermicompost plus NPS fertilizer, and [87] showed that combining inorganic fertilizer with compost maximized cob length. Moreover [72], also found the greatest cob length from NPSZnB, vermicompost, and lime, with shortest cobs in unfertilized controls. Similarly [50], demonstrated that blending NPSZnB fertilizer with farmyard manure increased ear length more than conventional fertilization, while [88] found manure combined with NP fertilizer enhanced panicle length in tef. Besides [37], also reported taller cobs with higher nitrogen rates.

#### Ear number per plant

The highest ENPP (1.63) was found in unified use of 240/138 kg N/P_2_O_5_ ha⁻¹ with 8 t BC ha⁻¹. Overall, combined inorganic and organic sources of nutrients applications produced higher ear numbers than sole applications or controls. The increased ear number likely results from improved nutrient uptake and soil conditions due to combined treatments, promoting better plant growth and ear formation. These results align with previous studies demonstrating that combined nutrient management enhances maize yield and ear number more than sole applications [37, 59, 89–92].

#### Grain number per ear

The increase in GNPE with combined inorganic and organic inputs likely results from better nutrient availability, promoting fuller grain filling compared to unfertilized plots. These results agree with previous studies showing that combined use of inorganic N/P₂O₅ nutrients with BC, lime, compost, or vermicompost improves grain number more than sole applications or controls [65, 72, 80, 93, 94].

#### Number of rows per ear

The enhanced NRPE with combined inorganic N/P₂O₅ and organic VC, and BC likely results from improved nitrogen availability through enhanced organic matter decomposition and root growth. This synergy positively influences physiological and biochemical processes during maize development, leading to more grain rows per ear. Our result is concurred with [57] found that the number of rows per ear ranged between 12 and 15 under draught stress and normal conditions in South Africa. Our finding also aligns with [93], who reported higher rows per ear with biochar plus nitrogen fertilizer compared to sole fertilizer or control. Similarly [95], demonstrated that combining organic matter with nitrogen fertilizer improves soil properties and maize yield. Additionally [96] and [97], found biochar enhances plant growth and yield by improving soil quality. Organic manure combined with inorganic fertilizer significantly increased grain rows per cob, with poultry litter plus fertilizer treatments performing best, while control had the fewest [98]. Increased rows per ear with combined bio-slurry and nitrogen fertilizer application [94].

#### Thousand grain weight

The TGW improvement likely results from the synergistic effects of inorganic N/P₂O₅ combined with organic VC and BC, which enhance nutrient availability, organic matter mineralization, and soil fertility, especially in acidic soils. This improves assimilate allocation to grains and overall yield components. This might be the availability of nutrients from inorganic N/P_2_O_5_, BC and VC sources of nutrients and synergetic effect could be the probable reasons for this highest TSW. These findings agree with previous studies showing combined organic and inorganic amendments increase grain weight and crop productivity [66, 99–101]. Similar benefits were reported in barley and wheat with integrated organic and inorganic nutrient management [79, 80, 102]. Likewise [103], noted significant grain weight and growth improvements in maize and wheat with VC, BC, and polymer combinations.

#### Biological yield

Due to rainfall variability across growing seasons, biological yield varied significantly, likely influenced by other climatic factors such as temperature, humidity, and solar radiation, as well as biotic stresses, soil nutrient dynamics, or management practices [104–106]. Our findings are also aligned with previous studies showing integrated organic and inorganic fertilizers significantly increase cereal biomass [27, 50, 80, 107, 108]. Nitrogen availability notably enhances maize biomass by prolonging growth stages [109].

#### Grain yield

The lower GY (4.4 t ha⁻¹) observed in the control (0.63 t CaCO_3_ ha⁻¹) treatment might be attributed to reduced soil nutrient content, nutrient mining, leading to limited nutrient availability for healthy and optimal plant growth. Our two years study results confirm the efficacy of combining BC and VC with N/P₂O₅ to enhance nutrient availability, uptake, and crop performance under acid-affected soils. Overall, the results suggest that ISFM practices can significantly improve maize grain yield and offer a sustainable strategy for managing soil fertility and productivity in Ethiopian highlands. The findings indicate that integrating BC with fertilizer applications can influence phosphorus dynamics in the soil, potentially enhancing phosphorus availability for crops. The other probable reasons might be due to BC’s liming effect and nutrient retention in acidic soils, VC’s slow-release nutrients and microbial activity, synergy leading to better nutrient use efficiency. However, the highest rates (T27) sometimes underperformed, which might be nutrient immobilization.

These findings align with earlier research highlighting the benefits of integrated soil fertility management (ISFM) in enhancing maize productivity. Similar improvements in yield from combined nutrient sources [110, 111]. In Northeast China [112], found that integrating organics with synthetic fertilizers sustained high yields and improved nutrient use efficiency.

In Ethiopia, ISFM has demonstrated substantial improvements in maize productivity across various agro-ecological zones. This is supported with [113] who found that the highest maize GY (6.07 t ha⁻¹) was achieved through the combined application of 10 t ha⁻¹ compost with 100/100 kg ha⁻¹ Urea and NPSB fertilizers, while the lowest yield (1.17 t ha⁻¹) occurred in unfertilized control plots. Likewise [27], found that the use of lime, either alone or in combination with biochar and mineral fertilizers, significantly enhanced soil pH and improved maize yield in acidic soils. In a similar vein [37], documented the highest grain yield (13.93 t ha⁻¹) under the highest nitrogen rate (360 kg ha⁻¹) and planting density (90,909 plants ha⁻¹), compared to the lowest yield (8.7 t ha⁻¹) obtained at the lowest nitrogen level (120 kg ha⁻¹) and planting density (53,333 plants ha⁻¹) in Jabitahinan district, Western Amhara Ethiopia.

Furthermore [101], observed that the application of 7.5 t ha⁻¹ vermicompost combined with 150 kg ha⁻¹ NPSB fertilizer yielded the maximum grain output (8.03 t ha⁻¹), whereas the untreated control plots produced the lowest yield (6.96 t ha⁻¹) in the Guto Gida district of Western Ethiopia. These findings collectively underscore the vital role of SFM in boosting maize yield under varying soil and climatic conditions. Additionally [11], found that continuous monocropping of maize at farmers field showed a declining trend in average yield at *Tyatya Kebele*, Burie Zuria district, with yields from plots treated with inorganic NPK (150-55-72 kg ha⁻¹) and 1 t compost ha⁻¹ decreasing from 7.94 t ha⁻¹ in 2016 to 7.75 t ha⁻¹ in 2017 and 5.94 t ha⁻¹ in 2018. In contrast, yields from untreated plots declined more sharply from 3.72 t ha⁻¹ in 2016 to 1.13 t ha⁻¹ in 2017 and 1.5 t ha⁻¹ in 2018.

### Cost benefit analysis for combined application of N/P_2_O_5_, BC and VC

The application of integrated inputs in T14 increased net benefits over their sole application by 32.92% (N/P₂O₅), 31.55% (BC), and 43.45% (VC), respectively. The findings support the use of localized organic inputs such as maize cob BC and VC to reduce reliance on expensive synthetic fertilizers. Vermicompost, for instance, can substitute up to 23.9 kg nitrogen per tonne, helping resource-poor farmers improve productivity affordably in the Burie district and similar agro-ecology.

Our study concur with [111] who reported profitability with 8 t ha⁻¹ BC and 50 kg NPS ha⁻¹ in Guto Gida district. Moreover [114], found the highest net benefit using 10 t ha⁻¹ farm yard manure and 69 kg N ha⁻¹. Similar integrated benefits were reported by [50, 80, 113, 115]. Earlier study by [116] also emphasized the role of site-specific NPK management combined with organic fertilizers in enhancing marginal returns. As a result, combined application of inorganic N/P_2_O_5_ (120/69 kg ha⁻¹), maize cob BC (4 t ha⁻¹) and VC (5.02 t ha⁻¹) was found economically feasible for maize production in acidic soils of Burie district and similar agro-ecologies.

## Conclusion and recommendations

The study demonstrated that integrated nutrient management through combined applications of inorganic N/P₂O₅ nutrients, BC, and VC significantly enhanced growth, yield traits, and yield of maize. The results of this study showed that the physiological, growth, and yield traits of the maize crop were considerably influenced by the mixed use of VC, BC, and inorganic N/P₂O₅ nutrients.

The longest time to reach 90% PM (174.73 days), tallest plants (320.50 cm), highest number of leaves per plant (NLPP, 17.45), longest ear length (20.39 cm), highest ears per plant (ENPP, 1.63), greatest number of grains per ear (GNPE, 647.11), highest number of rows per ear (NRPE, 15.03), thousand grain weight (TGW, 425.18 g), biological yield (BY, 27.87 t ha⁻¹), and grain yield (GY, 12.13 t ha⁻¹) were all recorded under the combined application of nitrogen, phosphorus pentoxide (N/P₂O₅), BC, and VC treatments (T27, T9, T24, T26, T25, T14, T9, T15, T25, and T24, respectively). In contrast, the lowest values for these parameters were observed in the untreated control plots.

The combined application of 120/69 kg N/P₂O₅ ha⁻¹+ 4 t BC ha⁻¹ + 10.04 t VC ha⁻¹ improved thousand grain weight by 44.83% compared to the control, and by 22.89%, 32.96%, and 27.44% compared to sole applications of N/P₂O₅, BC, and VC, respectively. Additionally, applying 120/69 kg N/P₂O₅ ha⁻¹ together with 8 t BC ha⁻¹ and 10.04 t VC ha⁻¹ increased grain yield by 63.73%, 29.50%, 40.97%, and 35.77% compared with the control and the sole application of maximum inorganic N/P₂O₅, BC, and VC, respectively. For T24, T14, and T17, grain yield was increased by 63.73%, 63.6%, and 62.5%, respectively, compared to the control. As a result, the most profitable treatment was 120/69 kg N/P₂O₅ ha⁻¹ + 5.02 t VC ha⁻¹ + 4 t BC ha⁻¹ (T14), which resulted in a net benefit of 289,124 ETB ha⁻¹ and a marginal rate of return (MRR) of 149.07%. In conclusion, integrated application of inorganic N/P₂O₅ nutrients, BC and VC at 120/69 kg ha⁻¹ + 4 t ha⁻¹ + 5.02 t ha⁻¹ is advised for the study area, as a mixed-use of the three nutrient sources improves the soil environment and increases production and productivity. The limitations of our study were; (i) the single-location, two-year nature of the study and the need for multi-location verification; (ii) the lack of post-harvest soil data, and (iii) the potential variability in the quality of produced BC and VC.

## Data Availability

The datasets analyzed during the current study are available from the corresponding author on reasonable request.

## Abbreviations

Al: Aluminum
ANOVA: Analysis of variance
BC: Biochar
BH: Bako hybrid
BY: Biological yield
C: Carbon
EL: Ear length
ENPP: Ear number per plant
ETB: Ethiopian Birr
GMC: Gravimetric moisture content
IF: Inorganic fertilizers
Ph: Power of hydrogen ion
PH: Plant height
RCBD: Randomized complete block design
TGW: Thousand grain weight
VC: Vermicompost
